# Data-Driven Prediction of Nanoparticle Biodistribution from Physicochemical Descriptors

**DOI:** 10.1021/acsnano.5c03040

**Published:** 2025-07-16

**Authors:** Jimeng Wu, Peter Wick, Bernd Nowack

**Affiliations:** † Empa, Swiss Federal Laboratories for Materials Science and Technology, Nanomaterials in Health Laboratory, Lerchenfeldstrasse 5, St. Gallen 9014, Switzerland; ‡ Empa, Swiss Federal Laboratories for Materials Science and Technology, Technology and Society Laboratory, Lerchenfeldstrasse 5, St. Gallen 9014, Switzerland

**Keywords:** nanoparticle pharmacokinetics, PBPK modeling, QSAR, biodistribution prediction, SSbD

## Abstract

Nanoparticles have gained significant attention in biomedicine, electronics, and environmental science due to their unique physicochemical properties, which critically influence their absorption, distribution, metabolism, and excretion behavior in biological systems. However, predicting nanoparticle biodistribution and pharmacokinetics remains challenging due to the complexity of biological systems and the reliance on animal-derived data for physiologically based pharmacokinetic (PBPK) modeling. To address these limitations, this study integrates PBPK modeling with quantitative structure–activity (QSAR) relationship principles and multivariate linear regression (MLR) to develop a predictive framework for nanoparticle biodistribution based solely on physicochemical properties, using biodistribution data from healthy mice. Focusing exclusively on nondissolvable nanoparticles, we employed Bayesian analysis with Markov chain Monte Carlo simulations to fit PBPK models and generate kinetic parameters. The MLR–PBPK framework demonstrated strong predictive accuracy for kinetic indicators (adjusted *R* ^2^ up to 0.9) and successfully simulated nanoparticle biodistribution across 18 experiments. Key physicochemical properties such as zeta potential, size, and coating were identified as the most influential predictors, while the core material and shape had lesser impacts. Despite its success, the model faced limitations in predicting concentration–time curves for certain nanoparticles, highlighting the need for expanded data sets and nonlinear modeling approaches. This study provides a robust, nonanimal alternative for nanoparticle risk assessment, advancing safe and sustainable by design (SSbD) frameworks and offering a valuable tool for early-stage nanoparticle evaluation and design.

## Graphical abstract

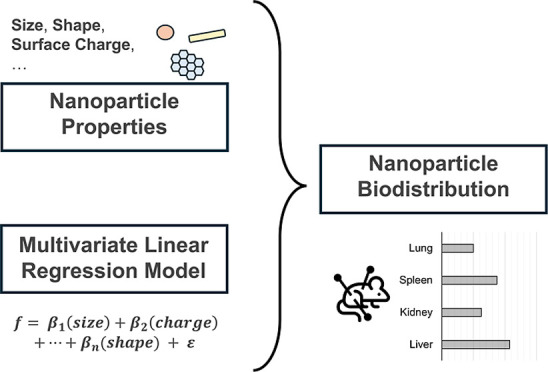

Nanoparticles have garnered significant attention in various fields, including biomedicine, electronics, and environmental science, due to their unique physicochemical properties.^1–3^ These properties—such as particle size, shape, and surface charge—play a critical role in determining their behavior in biological systems, particularly in terms of absorption, distribution, metabolism, and excretion (ADME).^4,5^ Their small size and ability to cross biological barriers can result in unintended bioaccumulation in different tissues, potentially causing toxic effects.^6,7^ For example, some studies showed that nanoparticles can accumulate in organs such as the liver, kidneys, and spleen, raising concerns about long-term exposure and possible toxicity.^8^ Understanding nanoparticle biodistribution is particularly important in the context of potential hazards associated with nanoparticles.

However, accurately predicting how nanoparticles interact with biological systems remains a significant challenge.^9^ The complexity of biological systems, alongside the various characteristics of nanoparticles, makes it difficult to fully understand their pharmacokinetics and tissue distribution. Physiologically based pharmacokinetic (PBPK) modeling has emerged as a valuable tool for predicting the ADME of nanoparticles within the body.^10,11^ PBPK models use mathematical equations to describe the movement of substances through different compartments of the body. PBPK models have been listed as one of the current quantitative support tools for the investigation of nanoparticle hazard assessment as specified in the OECD guideline^12^ and under REACH.^13^ A significant limitation of these models is that several critical kinetic parameters such as uptake rate and release rate constants are difficult to measure experimentally and are typically obtained by fitting animal experimental data.^14–16^ Consequently, the PBPK models rely heavily on animal studies, and the model simulation is usually limited to one type of nanoparticle, limiting the capability to extrapolate across different nanoparticle types. Given these challenges, there is a growing need for alternative, data-driven approaches that could reduce reliance on *in vivo* testing while maintaining predictive capability. This is particularly relevant in the context of safe and sustainable by design (SSbD),^17^ where nanoparticle assessment is hampered by the scarcity of available tools that are able to predict the hazards of nanoparticles using just nanoparticle properties.^18–20^ The concept of SSbD, promoted by the European Commission, aims to ensure that materials and products are designed with minimal environmental and health risks throughout their lifecycle. SSbD emphasizes early-stage hazard screening to avoid costly redesigns or regulatory issues later in development. Animal-free approaches are increasingly proposed as promising solutions to support SSbD.^21^ Within this context, computational models such as quantitative structure–activity relationship (QSAR) and PBPK modeling are encouraged as tools to predict biological behavior from material properties, thus reducing the reliance on *in vivo* testing.

Recent advancements in nano-QSAR development^22^ hold potential for overcoming the above-mentioned limitations. Recently, machine learning models have been trained based on the chemical structure and administered dose for drugs to predict pharmacokinetic parameters (*e.g.*, *C*_max_—the maximum concentration, AUC—the area under the curve, representing the integral of the concentration of a substance as a function of time) as well as time–concentration pharmacokinetic profiles.^23,24^ Similar models have also been extended to nanoparticles used in tumor delivery, based on nanoparticle properties and administration protocols.^25,26^ Few studies have also explored not just biokinetic indicators but also the PBPK model parameters. We have found one study^27^ that combined deep neural networks based on QSAR with PBPK modeling to predict delivery efficiency as well as the PBPK parameters of nanoparticles to tumors in mice. However, these models remain inadequate for evaluating nanoparticle risks in healthy biological systems, highlighting a critical gap for future application.

Theoretically, nano-QSAR models can generate nanoparticle-specific pharmacokinetic parameters based on their physicochemical properties, which can then be used as input for PBPK models. This approach allows for the prediction of nanoparticle biodistribution and pharmacokinetics without the need for extensive animal experiments, presenting a nonanimal alternative to facilitate nanoparticle SSbD research.

In this study, we focused exclusively on nonsoluble nanoparticles to isolate the impact of intrinsic physicochemical particle properties on biodistribution in healthy mice. Dissolvable nanoparticles (*e.g.*, Ag, ZnO) introduce confounding factors such as dissolved metal ions, which alter uptake mechanisms and necessitate distinct pharmacokinetic frameworks.^28,29^ By limiting the nanoparticle types to nonsoluble particles, we were able to focus more precisely on the influence of original properties on the nanoparticle’s biodistribution and pharmacokinetic behavior.

The objective of this study was to integrate PBPK modeling with QSAR principles and mathematical approaches to build a model to predict the pharmacokinetics of different nanoparticles based on their physiochemical properties. Using biodistribution data from healthy mice, we developed a multivariate linear regression (MLR) model to link nanoparticle kinetic parameters derived from biodistribution studies to their physicochemical properties. Bayesian analysis with Markov chain Monte Carlo (MCMC) simulation was utilized for the PBPK model fitting and kinetic parameter generation. Compared to traditional PBPK models, this new MLR–PBPK framework offers a significantly enhanced computational platform for predicting nanoparticle delivery efficiency without the need for animal-derived training data sets. This advancement has the potential to greatly improve the safety assessment of various types of nanoparticles, providing a robust nonanimal alternative for nanoparticle risk assessment and development.

## Results

### Analysis of the Nanoparticle Properties within the Data Set

The physicochemical properties used to characterize each published nanoparticle biodistribution experiment include six features: core material, particle shape, surface coating, zeta potential, hydrodynamic size, and dosing regimen. As shown in Table 1, more than half of the nanoparticles are spherical. In addition, polyethylene glycol (PEG) is the dominant coating type across the data set. The zeta potential category has a high level of missing data, with over half of the entries lacking values, leading to a “no info” designation.

**Table 1:** Summary of Biodistribution Studies in Healthy Mice Used for the Modeling^a^

experiment ID	nanoparticle	hydrodynamic size (nm)	size (nm)	zeta potential (mV)	shape	coating	maximum measurement time (h)	dose (mg/kg)	reference
1	iron oxide	29		–39	spherical	ethylenediaminetetraacetic acid (EDTA)	0.5	5	Sun et al., 2016 ^51^
2	iron oxide	41	5		spherical	dextran	48	4	Shanehsazzadeh et al., 2013 ^52^
3	SiO_2_		20		spherical	amino groups	720	10	Xie et al., 2010 ^53^
4	SiO_2_		80		spherical	amino groups	720	10	Xie et al., 2010 ^53^
5	Au	12	4		spherical	polyethylene glycol (PEG)	4320	0.85	Cho et al., 2010 ^54^
6	Au	23	13		spherical	PEG	4320	0.85	Cho et al., 2010 ^54^
7	Au	100	100		spherical	PEG	4320	0.85	Cho et al., 2010 ^54^
8	Au	34.6	6.2	–5.49	spherical	PEG	2160	3	Li et al., 2018 ^55^
9	Au	55.5	24.3	–6.53	spherical	PEG	2160	3	Li et al., 2018 ^55^
10	Au	77.1	42.5	–5.7	spherical	PEG	2160	3	Li et al., 2018 ^55^
11	Au	82.6	61.2	–7.36	spherical	PEG	2160	3	Li et al., 2018 ^55^
12	Au	27.6	13		spherical	PEG	168	0.85	Cho et al., 2009 ^43^
13	Au	27.6	13		spherical	PEG	168	4.26	Cho et al., 2009 ^43^
14	GO	20			sheet, single layer	PEG	1440	20	Yang et al., 2011 ^56^
15	GO	243	300		sheet, single layer		3	1	Liu et al., 2012 ^57^
16	GO	914	3000		sheet, single layer		3	1	Liu et al., 2012 ^57^
17	TiO_2_	352	20	–13.2	rod shape	amino groups	720	10	Xie et al., 2011 ^44^
18	TiO_2_	220	15		rod shape	hydrated amorphous silica	720	60.4	Sugibayashi et al., 2008 ^45^

a This table summarizes selected physicochemical characteristics and experimental details for different biodistribution experiments. The columns provide information on the following: experiment ID: a unique identifier for each nanoparticle experiment; nanoparticle type; hydrodynamic size: size of a nanoparticle in medium (nm); size: the primary nanoparticle size (nm); zeta potential, shape, and coating were also reported if available; maximum measurement time (h): the maximum duration (in hours) over which nanoparticle concentrations were measured post-injection; dose (mg/kg): the administered dose of nanoparticles (in milligrams per kilogram of body weight).

### Evaluation of the MCMC Fitting Results

The generalized PBPK model successfully simulated the biodistribution of nanoparticles across a wide range of published data sets, with simulations extending up to 4320 h (corresponding to 180 days) postinjection. Convergence was confirmed for every experiment using the calculated *R*-hat values as shown in Table S4, ensuring that the simulation chains reached a stable distribution. This model provides valuable insights into both short- and long-term nanoparticle concentration–time profiles within tissues, offering a perspective on time-dependent organ delivery efficiency that is challenging to achieve in traditional animal studies. In Figure 1, the results of the MCMC-fitted PBPK model are illustrated with two example experiments.

**Figure 1:**
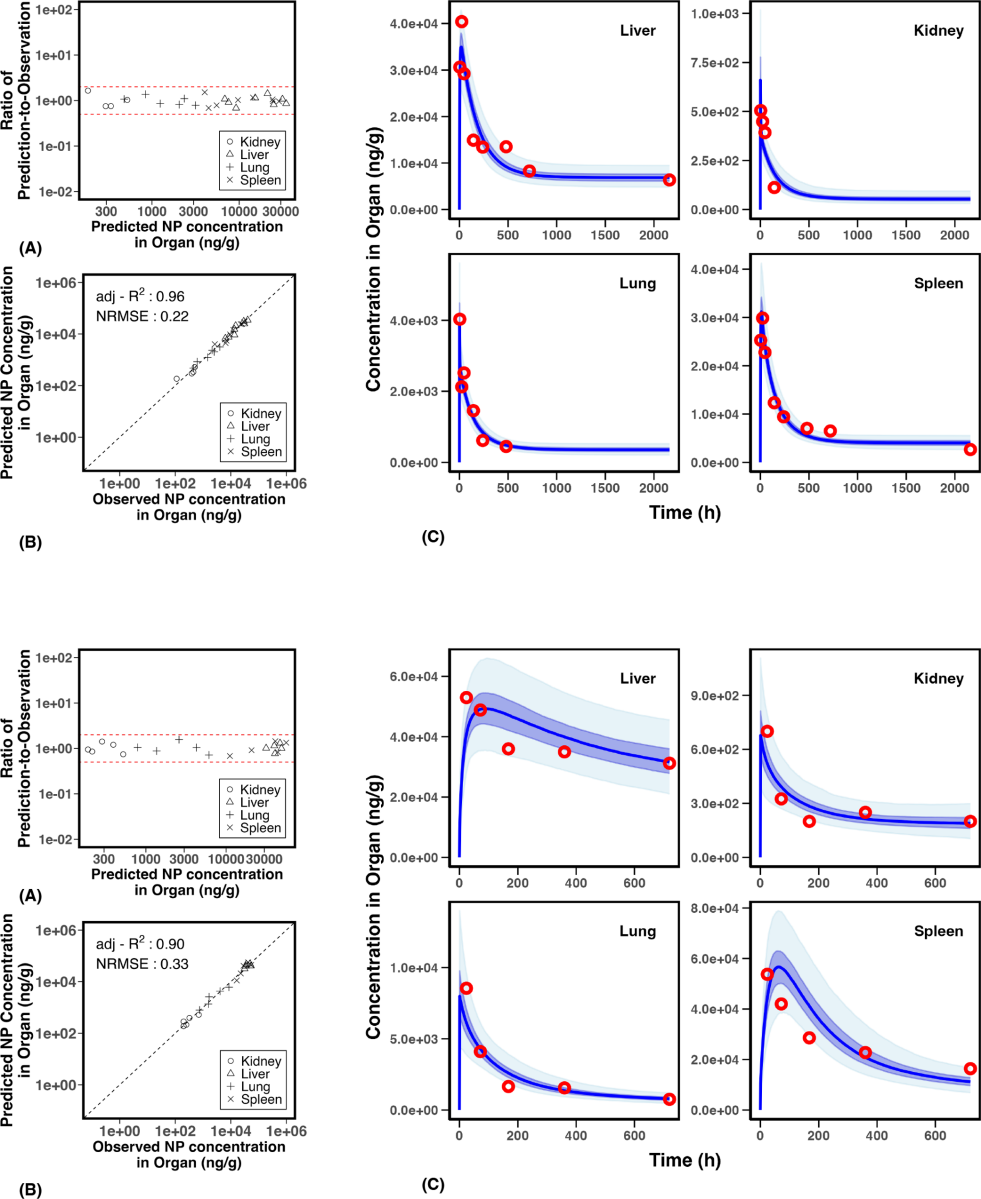
Examples illustrating MCMC results for experiments 11 (top) and 17 (bottom). The panels display (A) the predicted-to-observed ratio versus model prediction plot, (B) the global evaluation of the PBPK model MCMC fit, and (C) comparisons of the predicted biodistribution curves generated from MCMC-fitted posterior parameters with observed data points. In the first plot, the dashed lines indicate the thresholds for a predicted-to-observed ratio greater than 2 or less than 0.5. In the second plot, the dashed black diagonal line represents the unity line, where observed and predicted values are equal. In the third plot, the blue curve shows the fitted time–concentration curve, with light and dark shading representing the interquartile range (25%–75%) and the 95% confidence interval, respectively. Red points mark the measured concentrations of nanoparticles in four organs.

When predicted concentrations were compared with observed data for every experiment, the model demonstrated good agreement, achieving an adjusted *R* ^2^ of 0.85 (Figure 2). Approximately 92% of the predictions fell within a 2-fold difference from observed values, which is the WHO model precision criteria.^30^

**Figure 2:**
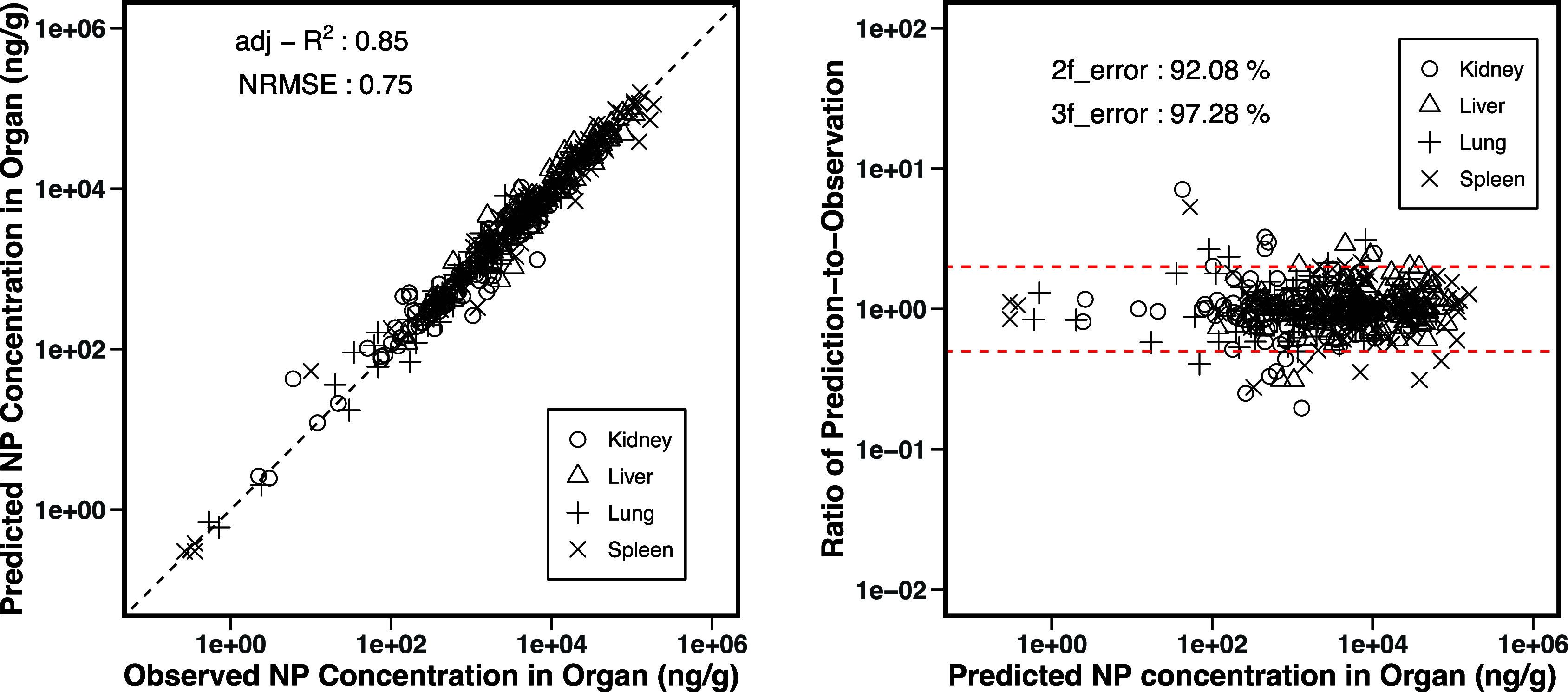
Global evaluation of the goodness of model fit of the concentration–time curve in four organs for every biodistribution case. (Left panel) Comparison of model calculations with observed data. (Right panel) The predicted-to-observed ratio versus model prediction results.

Lower concentration ranges (10–10^2^ ng/g) are generally linked to later time points (over 24 h), whereas higher concentration ranges (10^2^–10^6^ ng/g) tend to correspond to earlier times, within the first 24 h, as shown in Figure S3. Model predictions showed greater variability at lower concentration ranges than higher ranges, but at higher ranges, the model showed larger deviations between predicted data points and observed values. In the fitting results for lower concentrations, the most notable deviation occurred in the kidney for experiment 14 (20 nm of graphene oxide), followed by the spleen in experiment 2 (41 nm of iron oxide). In these cases, the model predicted a kidney concentration of 50 ng/g, while the observed value was only 6 ng/g at 72 h, and in the FeO study, the model predicted 53 ng/g for the spleen, compared to the observed 5 ng/g at 48 h. Despite these large predicted-to-observed ratios, the absolute deviation remained relatively small at around 45 ng/g, compared to the total concentration range of up to 10^6^ ng/g, proving a good calculation ability.

At higher concentration levels, the largest deviation was observed in the kidney during experiment 13 (27.6 nm high-dosage Au), where the model predicted a concentration of 1305 ng/g, while the observed concentration was 6624 ng/g at 0.08 h. A similar discrepancy occurred in experiment 12 (27.6 nm low-dosage Au), where the model predicted 1044 ng/g, compared to the observed 261 ng/g at 0.08 h. In both experiments, the model struggled to capture the rapid concentration increases in the early stage but effectively simulated the overall trend.

Case-by-case evaluations, as detailed in Table S5 and Figure S4 in the Supporting Information, show that adjusted *R* ^2^ values typically range between 0.7 and 1, except for experiment 14 (20 nm GO). Its lower value (adjusted *R* ^2^ = 0.48) was attributed to the limited number of early phase measurement points. In contrast, experiment 2 (41 nm iron oxide), with more frequent early phase measurements, achieved a higher adjusted *R* ^2^ of 0.87, as the additional data points helped smooth deviations at higher concentrations. Every experiment was proved to be convergent given the calculated *R*-hat assessment to ensure that the chains in the simulation have reached a stable distribution.

### MCMC Model Validation

The validation results in Figure S5 show that the PBPK model could accurately predict the kinetic concentration curves within the time frame of the provided data set. However, parameters derived from the shorter measurement time could not reliably predict outcomes for the longer-duration study, as shown in the lower panel in Figure S5. This discrepancy indicates that the information available from short-term kinetic data may not sufficiently constrain parameters governing slower biological processes, such as nanoparticle redistribution or clearance occurring at later time points. As a result, models trained on short-duration studies may underperform when extrapolated to longer temporal ranges. This supports the robustness and reliability of the MCMC model, as parameters fitted to one study were consistent and able to predict outcomes in an independent study with similar conditions. These findings indicate that the fitted parameters reflect underlying physiological processes, but the model’s applicability is limited to data sets within the temporal range of the training data.

### Analysis of the Parameters of the MCMC-Fitted PBPK Model

Figure 3 shows a heatmap of the fitted PBPK model parameters across all experiments, highlighting clustering tendencies. Iron oxide experiments exhibited the most unique PBPK model parameter values, with the darkest colors occurring most frequently. Au was the most highly represented nanoparticle type in the data set, forming a tight cluster with consistent PBPK model parameter values across all cases. GO and iron oxides also exhibited tight clustering, which suggests that the nanoparticle core material strongly influences the PBPK model parameter distribution. The GO study (experiment 14) that aligned with Au includes the same PEG coating, whereas the other GO studies lack such modifications, indicating the importance of coating.

**Figure 3:**
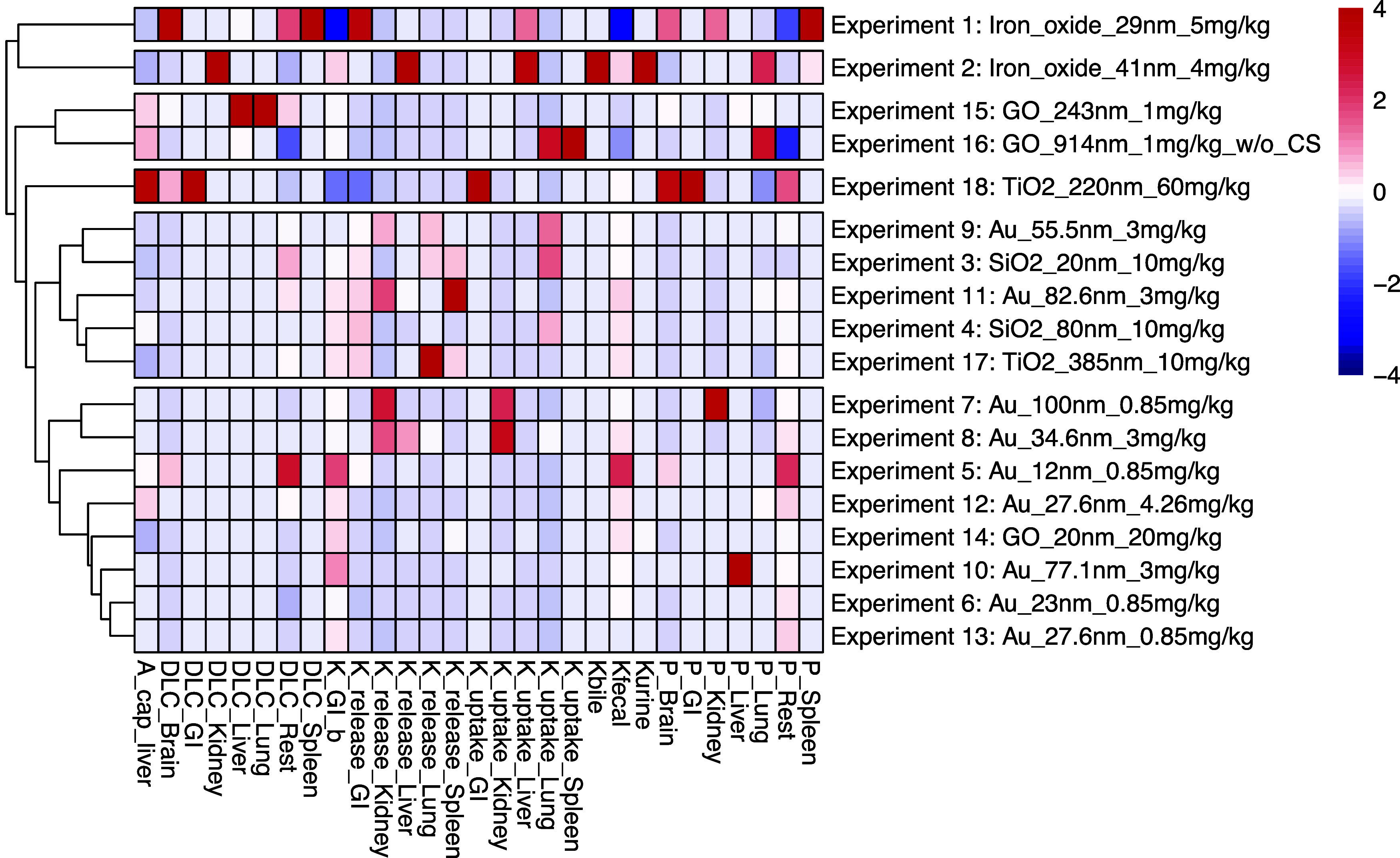
Heat map plot of the mean parameter values from each nanoparticle study posterior parameter distribution. The plot identifies similarities between the parameters for each nanoparticle case. Each column represents one parameter, and each row represents one nanoparticle study. Definitions of all parameters are available in Table S2 in the Supporting Information. Cell colors are coded based on the deviation of a parameter value in one study compared to all nanoparticle studies for specific parameters; darker colors represent more different values. Rows are sorted by hierarchical clustering (Canberra distance, ward.D2 linkage^31^).

### MLR—Predicting the PBPK Model Parameters

Twenty-nine endocytosis-related parameters were predicted among all of the study cases using MLR using the six considered nanoparticle characteristics. The overall MLR model prediction accuracy of the PBPK model parameters was good, achieving an adjusted *R* ^2^ of up to 0.92, as shown in Figure 4, demonstrating its effectiveness in capturing general nanoparticle biodistribution trends in four organs.

**Figure 4:**
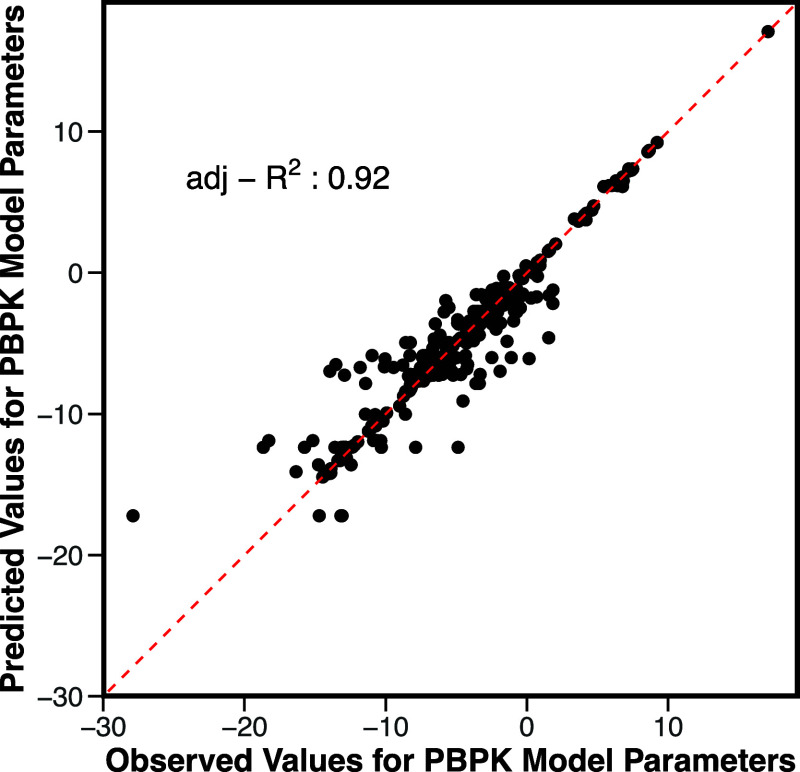
Validation of PBPK model parameters calculated from the MLR model. This figure illustrates the accuracy of predicted parameters when derived from the MLR model compared to the Bayesian MCMC fitting-derived value.

The individual PBPK model parameter prediction performance and the generated detailed MLR equations are given in Table S6. Most of the parameters have an accuracy higher than 0.7, though a few exhibited lower accuracies due to either multimodal distributions, as shown in Figure S6, or narrow value ranges, limiting the model’s prediction ability. An example of a parameter that only showed a narrow range is DLC_Rest (the value is either 14.1 or 14.2). Consequently, predicted values were confined to this range and closely matched the observed values. However, due to the limited variability, the *R* ^2^ value appeared low, despite the small absolute error. This shows the challenge of evaluating model performance for parameters with minimal variation. The poorly predicted PBPK model parameters suggest that while the overall model performs well, refinement or additional data may be needed to improve the accuracy of predictions for specific parameters.

Using the PBPK model parameters derived from the MLR models, we generated the corresponding time–concentration curves and compared them with observed time–concentration curves. The comparison of these generated biodistribution curves with observed data points yielded an adjusted *R* ^2^ of 0.43, as shown in the Supporting Information Figure S7, reflecting moderate predictive accuracy. Notably, 65% of the predicted data points fell within a 3-fold error margin of the observed values. Experiment-by-experiment accuracy analysis, detailed in Table S7, revealed a variability in performance. While some cases achieved high *R* ^2^ values of up to 0.9, others had *R* ^2^ values below 0.6.

### Predicting Kinetic Indicators Using MLR

The overall prediction accuracy for the kinetic indicators, as illustrated in Figure 5, is around 0.93, indicating a high level of predictive accuracy. The adjusted *R* ^2^ values were 0.95, 0.93, 0.89, and 0.94 in DE_24_, DE_168_, ARA, and *C*_max_, as shown in Figure S8, respectively. The generated MLR equations and accuracy for each kinetic indicator are summarized in Table S8. These results reveal that almost all kinetic indicators demonstrate an adjusted *R* ^2^ greater than 0.7.

**Figure 5:**
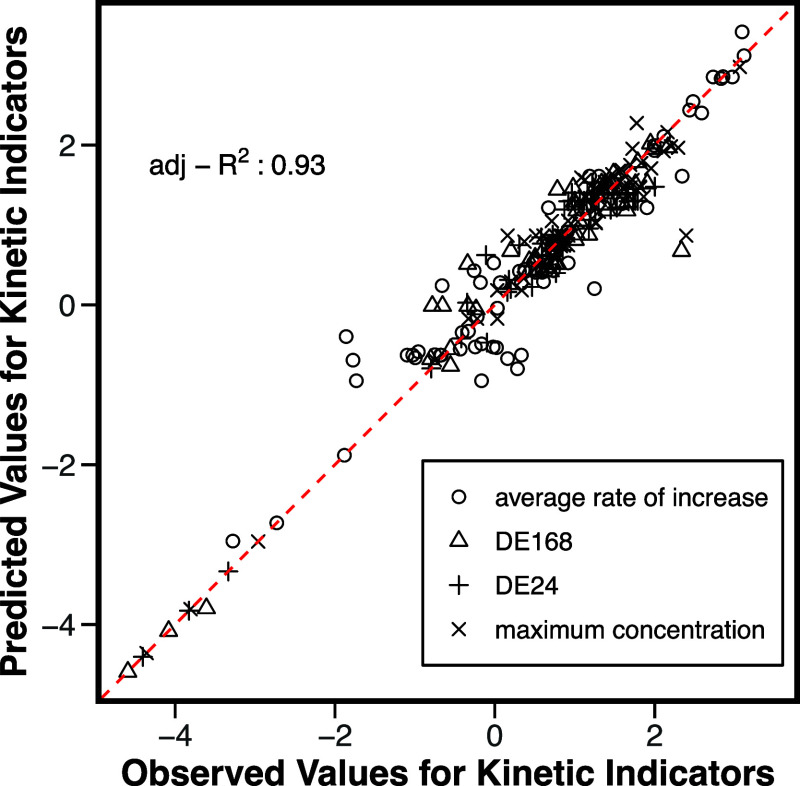
Validation of kinetic indicators (DE_24_, DE_168_, ARA, and *C*_max_) calculated from the MLR model. The figure shows the accuracy of the predicted kinetic indicators derived from the PBPK MLR model (*y*-axis) compared to the observed data (*x*-axis).

### Importance of Material Properties for Predicting Model Parameters

An analysis of the predictor frequency across all MLR models, shown in Figure 6, highlights the order of importance of all nanoparticle properties. Zeta potential, size, and coating stand out as the most influential predictors in both cases. Interestingly, despite zeta potential having only four categorical groups with less diversity than the other properties, it remains the most recurrent predictor. In contrast, shape and core material appear to be less significant, ranking lower than the other properties.

**Figure 6:**
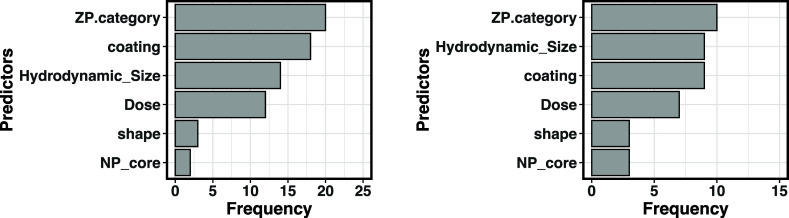
Frequency of nanoparticle properties occurrence in the best-predicted MLR equations. The left panel represents the MLR prediction for PBPK model parameters. The right panel represents the MLR prediction for kinetic indicators.

## Discussion

Over the past decade, PBPK models have been increasingly applied in the nanoparticle field. However, they are typically tailored to one specific nanoparticle.^32,33^ Our model represents a generalized approach capable of integrating data analysis with biodistribution databases. The MLR–PBPK model has been developed using five different types of nanoparticles—Au, iron oxide, TiO_2_, GO, and SiO_2_—providing insights into how key nanoparticle properties such as zeta potential, coating, and core material influence biodistribution. The predicted kinetic indicators from this MLR–PBPK model strongly correlate with the model fitting results, particularly in predicting delivery efficiency. While the model generally predicts nanoparticle kinetic indicators well, its predictions for the concentration–time curve (PBPK model parameters) were less promising.

The MLR analysis revealed that coating and zeta potential significantly influence the kinetic parameters, consistent with previous studies highlighting the importance of size, surface charge, and other physicochemical properties on nanoparticle kinetics and biodistribution.^34–36^ These observations indicate that surface chemistry might be the more important factor affecting nanoparticle biodistribution behavior compared with the core material. This observation aligns with the established understanding that surface chemistry plays a crucial role in determining nanoparticle behavior in biological environments, especially for insoluble nanoparticles.^36^ The nanoparticle core material ranked as the least significant factor, likely because most nanoparticles in our data set were coated, obscuring the core material’s influence on nanoparticle behavior. This does not eliminate the possibility that the core material may affect the nanoparticle transport behavior under different circumstances. The same situation holds for shape, as the distribution of shape is strongly dominated by spherical nanoparticles, with only a few instances of rod- and sheet-shaped nanoparticles.

Despite exhibiting different biodistribution behaviors, nanoparticles yielded similar PBPK model parameter outcomes due to the predictors selected in our study. This limitation is further highlighted in the nanoparticle characteristics similarity matrix (Figure S9), which clusters certain nanoparticles as similar based on their characteristics. However, significant variability in PBPK model parameters remains within these nanoparticle clusters, underscoring the need for more diverse or refined predictors to accurately capture these differences. For example, while one iron oxide nanoparticle experiment achieved an accuracy of 0.7 using the MLR–PBPK model, another iron oxide case with seemingly similar properties achieved an accuracy of only 0.2. A similar discrepancy was observed with GO nanoparticles; one experiment with a 914 nm nanoparticle size achieved 0.94 accuracy, whereas the other two experiments performed below 0.5. This suggests that the nanoparticle property data may not be sufficient to differentiate between similar nanoparticles, resulting in the model predicting identical outcomes for experiments that differ in reality.

The PBPK model parameters with multimodal distributions in our data set are not well predicted by the model, reflecting relationships that a linear approach cannot adequately capture. MLR models work well under linear assumptions but tend to perform poorly with data that show multimodal relationships.^37^ This may also be due to the small data set used for the MLR model, with only 18 nanoparticle experiments to predict 29 PBPK model parameters, which limits the MLR ability to fully capture the complex relationships between nanoparticle properties and PBPK model parameters. The small data set size also prevented formal validation of the model, raising concerns about potential overfitting.^38^

Nonetheless, the current framework offers promising potential for predicting the pharmacokinetics of new nanoparticle formulations beyond those included in this study. For a new formulation, the corresponding descriptors can be used as input into the trained MLR–PBPK pipeline to estimate biodistribution and pharmacokinetic behavior. However, when new nanoparticles include features not represented in the training data—such as novel coatings, targeting ligands, or unusual materials—prediction accuracy may decline due to extrapolation beyond the learned parameter space. In such cases, adding new formulations to the data set and retraining the model would enhance reliability.

While this study focused on nondissolvable nanoparticles without conjugated therapeutic agents, it is important to note that drug-conjugated nanoparticles present additional modeling challenges. Modeling such systems would require accounting for drug release kinetics, binding stability, and changes in surface properties that affect biodistribution and clearance. Key PBPK model parameters, such as endocytosis rates, tissue distribution, and elimination pathways, may also need to be revised correspondingly.

## Study Limitations

Although the MLR–PBPK model offers interpretable insights into nanoparticle biodistribution, it has its own limitations. First, the data set used in this study is relatively small and includes a narrow range of nanoparticle types. This constraint limits the model’s generalizability to broader nanoparticle formulations. Additionally, we acknowledge that MLR may not fully capture the interaction effects inherent in nanoparticle–biological system interactions. This shortcoming is particularly evident in PBPK model parameters with multimodal distributions, where the model exhibited reduced predictive accuracy. Third, biological complexities that influence biodistribution—such as protein corona formation—are not explicitly modeled. These can alter the physicochemical properties of the nanoparticles and further influence their pharmacokinetics significantly^39^ but are not captured in static descriptors such as size or zeta potential. Likewise, the framework is limited to nondissolvable, nondrug-conjugated nanoparticles and does not incorporate drug or ion release kinetics. Finally, due to the limited data set size, formal model validation (*e.g.*, independent test sets) was not feasible, raising the potential for overfitting, especially given the high dimensionality of the PBPK parameter space.

To address current limitations and improve the model’s performance, several steps on both the data and model levels are recommended. Expanding the data set, once publicly available, to include a broader range of physicochemical properties will reduce the current simplification of nanoparticle–PBPK relationships and improve the model’s ability to distinguish between similar nanoparticles. Increasing the size of the data set could also help to enhance the model’s generalizability. Additionally, *in vitro* experiments could be designed to focus on poorly predicted PBPK parameters, enabling refinement of the model and avoiding the implementation of animal biodistribution studies. While MLR was selected due to its interpretability and stable performance on small data sets, aligned with our focus on reducing animal experimentations, it may not fully capture the complexity of nanoparticle–biological interactions. Incorporating nonlinear modeling approaches or hybrid models, such as gradient boosting,^40^ support vector machines, or random forests, could better capture the complex behavior of nanoparticles, especially for PBPK model parameters with multimodal distributions.

## Conclusions

In conclusion, this study marks a significant advancement in nanoparticle biodistribution modeling through the development of a generalized MLR-assisted PBPK model. This model offers a novel approach to predict nanoparticle delivery efficiency in mice using only physicochemical characteristics of the nanoparticles, addressing a key challenge in SSbD, the scarcity of predictive tools capable of assessing material hazards using only material-specific properties, and minimizing the reliance on additional animal studies.

Despite its promising performance in predicting kinetic indicators, to get a better prediction for biodistribution kinetics, limitations such as the size and complexity of the current data set highlight the need for further refinement. Expanding the data set, exploring nonlinear models, and developing corresponding *in vitro* experiments will be crucial for enhancing the model’s accuracy. This incremental approach, where preliminary success paves the way for more complex predictions, suggests that with additional data, the model’s potential for capturing the full range of nanoparticle biodistribution behavior could be realized. Importantly, the modular structure of the PBPK model allows for cross-species extrapolation by replacing species-specific physiological parameters such as organ volumes and blood flow rate. These adjustments could be made using established physiological databases.^41^

Through developing and sharing the model under FAIR principles, we aim to enable further community-driven extensions. The model can be retrained or expanded as new data become available, including from disease models, novel nanoparticle types, and human studies. Hosting it on an open-access platform will support reuse and transparency, fostering its broader adoption in regulatory settings.

As a screening tool, it offers a more accurate and efficient method for designing and evaluating nanoparticles. Moreover, the model holds the potential for broader applications, such as cross-species extrapolation in PBPK modeling by adjusting physiological parameters, increasing its relevance in toxicity research and nanoparticle evaluation across various biological systems. In research, it reduces the reliance on time-consuming and ethically sensitive *in vivo* studies by enabling early-stage, *in silico* screening of nanoparticle formulations. For industry, the model can serve as a rapid decision-support tool during product development. In regulatory contexts, the model presented in this work could be of high value as an early innovation tool to be used within the SSbD assessment of materials, which could be applied during the initial phases of material design and safety assessment, before large-scale production. This tool helps people identify potential risks early on, offering a more efficient, data-driven approach to understanding and predicting nanoparticle biodistribution.

## Methods and Data

### Study Design and Overview

By leveraging a data set of nanoparticle physicochemical properties alongside PBPK model parameters derived from mouse biodistribution data, an MLR model was trained to identify relationships between nanoparticle properties and their biodistribution behavior in biological systems. The workflow followed three main stages as shown in Figure 7: (A) data collection, (B) nanoparticle delivery efficiency and PBPK model parameter estimation, and (C) MLR model development.

**Figure 7:**
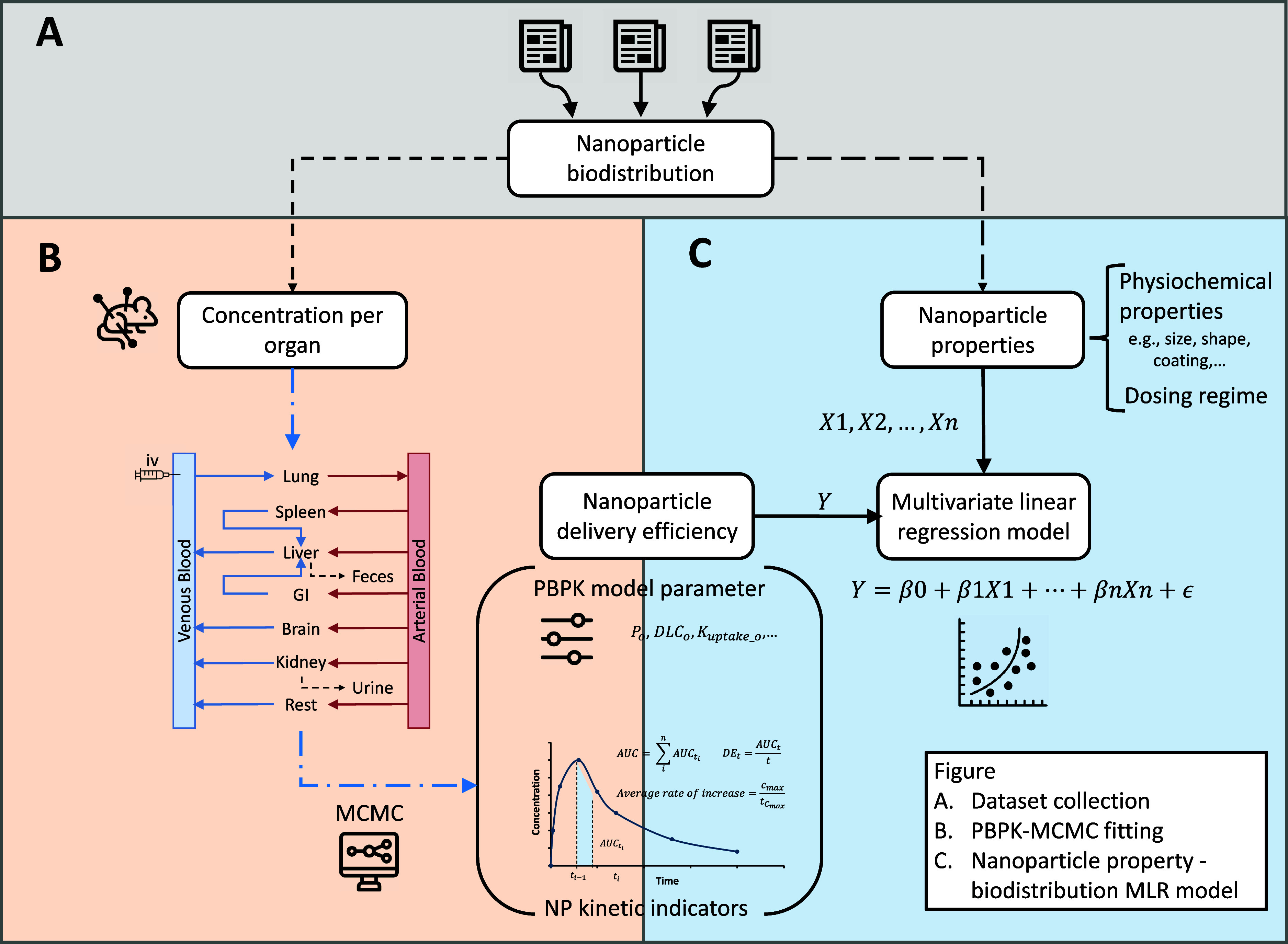
Scheme of the study framework. (A) The first step involved collecting a comprehensive data set containing information on nanoparticle physicochemical properties, dosing regimens, and observed time–concentration mouse biodistribution data points. (B) Using the collected data set, time–concentration data points were fitted with the PBPK model via MCMC simulations. The fitting process generated PBPK model parameters and corresponding time–concentration curves, from which essential kinetic indicators (*e.g.*, area under the curve, maximum concentration) were derived. (C) An MLR model was employed to establish the relationship between the nanoparticle physicochemical properties and their biodistribution characteristics, including both kinetic indicators and PBPK model parameters. Icons adapted from Freepik, HAJICON, and Creatype via www.flaticon.com.

### Nanoparticle Biodistribution Data Collection and Preprocessing

We selected biodistribution studies from a review paper^42^ which compiled literature on different nondissolved nanoparticle biodistribution in mice until 2020. For the time–concentration curve generation, we applied stringent exclusion criteria to the initial 115 studies to ensure the data suitability for PBPK modeling. Studies were included only if they (1) reported at least two time points (88 studies remaining), (2) were conducted on healthy mice (21 studies remaining), and (3) were not using radiation intensity as a concentration unit (7 studies remaining). From the initial 115 studies, we selected 7 animal biodistribution studies, supplemented by 3 additional studies^43–45^ identified through literature search in order to incorporate TiO_2_ and to include one gold nanoparticle experiment under the same conditions as one already collected data set for validation, resulting in a total of 10 studies encompassing 18 distinct nanoparticle biodistribution experiments as summarized in Table 1. Intravenously administered nanoparticles circulate in the blood until they are cleared from circulation and eliminated from the body by two main mechanisms: (i) renal elimination and (ii) hepatobiliary elimination,^46,47^ and mostly accumulate in the organs of the reticuloendothelial system, such as the liver, spleen, and lungs.^48–50^ Based on the physiological mechanisms and data availability, four organs were selected for subsequent PBPK model fitting: kidney, liver, spleen, and lung. After gathering the data set, all data were normalized into the same units, ng nanoparticle per organ weight (ng/g).

We selected, based on importance and data availability, six nanoparticle characteristics: core material, coating, shape, zeta potential, hydrodynamic size, and dose. The zeta potential was categorized as follows: negative (below −10 mV), positive (above 10 mV), neutral (−10 to 10 mV), and “no information”. When the hydrodynamic diameter was unavailable, the primary particle size was used as a substitute. The variables were classified into two types: categorical data (zeta potential, core material, shape, and coating) and numerical data (hydrodynamic size and dose), as outlined in Supporting Information Table S1.

### PBPK Model Structure for Nanoparticle Biodistribution

The mouse PBPK model, depicted in Figure S1, consisted of eight compartments: blood, lung, liver, kidney, spleen, GI tract, brain, and remaining tissues, which are essential to describe the pharmacokinetics and pharmacodynamics of nanoparticles with an injection pathway.^58^ For the lungs, spleen, liver, and kidneys, each organ was subdivided into capillary blood, tissue interstitium, and endocytic/phagocytic cells (PCs). The GI tract compartment includes capillary volume, tissue, PCs, and lumen sections, while the other organs consist of capillary blood and tissue interstitium.

PCs represent a wide variety of phagocytic cells, including what other investigators have called reticuloendothelial system cells, mononuclear phagocyte system (MPS) cells,^59^ or organ-specific cells, such as Kupffer cells in the liver, splenic macrophages, mesangial cells in the kidneys,^60^ etc. Based on their physiological locations, the mononuclear phagocyte system (MPS) compartment is present in the interstitium for organs such as the lung and kidney. It has also been added in the vascular compartment of the liver and spleen, as the majority of Kupffer cells^61^ and splenic macrophages^62^ have direct access to particles in the vascular compartment.

As the nanoparticles modeled in this study are nondissolvable, elimination occurs solely through excretion. Specifically, renal excretion via the kidneys (urine) and hepatobiliary clearance via the liver (feces) were incorporated as the elimination pathways, consistent with the behavior of nondissolvable nanoparticles that remain chemically stable in biological environments.

The PBPK model parameters were classified into two categories: physiological parameters of the organism and parameters related to nanoparticle endocytosis. The physiological parameters were sourced from the literature and are detailed in Table S2. The PBPK model included 29 endocytosis-specific parameters, which include organ-specific permeability coefficients (DLC_o_), tissue-to-blood distribution coefficients (*P*_o_), uptake (*K*_o_^uptake^) and release (*K*_o_^release^) rates by phagocytic cells, and clearance-related constants (*e.g.*, *K*_GI_^b^, *K*_bile_, *K*_urine_, and *K*_fecal_), as well as the uptake capacity of the liver tissue (*A*_Liver_^cap^). Initial values for endocytosis-specific parameters were determined by fitting data from a study on 23 nm gold nanoparticles (experiment 6), which had the longest observation period and the most comprehensive data set. These values are listed in Table S3. Detailed mathematical formulations for nanoparticle uptake and release processes are provided in the Supporting Information.

### PBPK Model Parameter Generation from Markov Chain Monte Carlo Fitting

The physiological parameters were kept the same during the whole fitting process, and the endocytosis parameters were fitted according to each experiment. To enhance the model performance during parameter optimization using the MCMC algorithm, a preliminary model calibration for the PBPK model for each experiment was conducted to obtain the initial value for the model parameters. First, a local sensitivity analysis was performed on all parameters using the R package FME.^63^ Parameters with a normalized sensitivity coefficient greater than 0.5 were typically selected for calibration. A manual selection of additional parameters was implemented, if needed. Then, the values for these selected parameters were estimated using the Levenberg–Marquardt algorithm^64^ based on observation data for each experiment.

The estimated parameters for each experiment were used as prior information for inclusion in the subsequent parameter optimization using the Bayesian MCMC method. The key idea of Bayesian statistics is to define unknown parameters as random variables, which is in contrast to the general approach in statistics, where parameters are defined as fixed but unknown constants. In Bayes’ theorem, prior knowledge about the parameters is updated with new experimental data in the so-called posterior distribution.^65^ Given the complexity of directly determining posterior distributions in nonlinear models with multiple parameters, MCMC methods, which enable estimation through sampling, have been used in pharmacokinetic modeling before.^66^ The core principle of MCMC involves sampling unknown variables along a Markov chain until they converge to a stationary posterior distribution, which is particularly useful for high-dimensional probability distributions. The details regarding the posterior probability distribution used in this study are explained in the Supporting Information.

To estimate the posterior distribution of the parameters, the Delayed Rejection Adaptive Metropolis sampling^67,68^ was employed for MCMC sampling. Four independent Markov chains were run, each for up to 600,000 iterations, with the first half designated as “burn-in” (*i.e.*, iterations for which the simulation had not converged yet) and the latter half used for output to assess convergence. We recognize that MCMC sampling, if not properly diagnosed, may lead to unstable parameter estimations and a reduction in model reliability. To ensure robustness and convergence in our parameter estimates, the convergences of the posterior parameter distributions sampled from the MCMC simulation were diagnosed by the potential scale reduction factor (*R*-hat), which measures the degree to which variance (of the means) between chains exceeds what one would expect if the chains were identically distributed. A convergence diagnostic *R*-hat value of 1.2 or less has been proposed as a criterion of acceptable convergence,^69^ representing a stable probability distribution.

All model simulations were conducted using R. The PBPK model was coded in the R package mrgsolve, while MCMC simulations were run within the R software package FME, which was developed particularly for nonlinear models and MCMC simulations. All model codes are open-source and are available in the Supporting Information and in GitHub.

### MCMC PBPK Model Result Evaluation, Validation, and Analysis

Using the obtained PBPK model parameters, we generated concentration–time curves for the four target organs. We then assessed the MCMC model fit by comparing the predicted and observed organ concentrations at measured time points. The determination coefficient (*R* ^2^) and the predicted-to-observed ratio were calculated to evaluate the model’s predictive accuracy against experimental data. According to the World Health Organization acceptance criteria, a predicted-to-observed ratio within a factor of 2 (*i.e.*, between 0.5 and 2) indicates an acceptable prediction outcome.^30^

A cross-comparison approach was used to validate the model, selecting two experiments (experiments 6 and 13) from separate studies that shared identical experimental parameters. These experiments involved nanoparticles with the same core material (gold), core size (13 nm), dosing regimen (0.85 mg/kg), and surface coating (thiol-terminated PEGs). The primary difference lay in the measurement duration, with one study offering a longer observation period than the other. To validate the model, parameters derived from fitting experiment 6 were used to calculate the time–concentration curve of experiment 13. The predicted outcomes were then compared with the results obtained by fitting parameters directly from experiment 13, and vice versa.

The distribution of generated PBPK model endocytosis-related parameters among all the experiments was first studied to detect if there is a certain pattern among different nanoparticle properties. The distribution of generated PBPK model parameters across all experiments was analyzed to investigate the potential patterns associated with different nanoparticle properties. We performed hierarchical clustering of the mean parameter values derived from the posterior distributions of each study. The clustering was based on the Canberra distance, which is sensitive to relative differences between low and high magnitude values.^70^ This is especially relevant in our case, where PBPK parameters span varying magnitudes due to biological variability. We used the Ward.D2 linkage method for hierarchical clustering.^71^ This method was chosen because it minimizes the total within-cluster variance and produces compact, homogeneous clusters. Compared with alternative linkage methods (*e.g.*, single, complete, average), Ward.D2 better preserves the global structure of the data when the number of clusters is unknown. This comparison allowed us to examine the similarity of biodistribution behavior (indicated by PBPK model parameters) among the experiments in the collected data set.

### Multivariate Linear Regression Model Development

Multivariate linear regression was used to investigate the relationship between the characteristics of nanoparticles’ biodistribution scenario and their kinetics and biodistribution behavior. Six key characteristics were selected to represent each nanoparticle case, as stated before. The fitted endocytosis-related PBPK model parameters and the selected kinetic indicators were treated as the predicted outputs. The kinetic indicators were derived from the fitted PBPK model and calculated from the corresponding time–concentration curves from the MCMC calculation.

Four kinetic indicators were selected to provide a comprehensive assessment of nanoparticle biodistribution, as shown in Figure S2 in the Supporting Information: (1) delivery efficiency at 24 h (DE_24_), (2) delivery efficiency at 168 h (DE_168_), (3) average rate of accumulation at maximum concentration (ARA), and (4) maximum concentration (*C*_max_). The delivery efficiency was calculated by dividing the area under the concentration–time curve by the respective time interval, providing a measure of nanoparticle persistence in the target organs. As the time to reach the maximum concentration was different for different nanoparticles, we therefore used the time when the maximum concentration (*t*_*C*_max__) occurred to normalize the maximum concentration (*C*_max_), representing the average accumulation rate for the nanoparticle (ARA). These kinetic indicators offered a robust understanding of nanoparticle biodistribution and transport behavior across various biological systems.

To ensure that all kinetic indicators and PBPK model parameters remained positive, a logarithmic transformation was applied. The relationship between the selected nanoparticle physicochemical properties (predictor variables) and the pharmacokinetic outputs (response variables) was modeled using eq 1:

1 $$Y=\beta_{0}+\beta_{1}X_{1}+\beta_{2}X_{2}+...+\beta_{n}X_{n}+\epsilon$$

where *Y* represents the predicted parameter (*e.g.*, kinetic indicators (DE_168_, ARA, ...) or PBPK model parameters); *X*_1_, *X*_2_, ..., *X*_*n*_ represent the nanoparticle physicochemical properties; β_1_, ..., β_*n*_ are the regression coefficients representing the influence of each nanoparticle property; and ϵ is the intercept term.

The coefficients (β) were estimated using the least-squares method, which minimizes the sum of the squared differences between the observed and predicted values of the response variable. This was implemented using statistical software R, which provides built-in functions for linear regression (function lm in R). The best subset model with the highest adjusted *R* ^2^ and lowest AIC/BIC values was selected as the final predicted equation.

The *R* ^2^ for both PBPK model parameters and kinetic indicators was computed to assess the fit of the MLR model. For the PBPK model parameters, we further evaluated the fit of the MLR model by comparing the concentration–time curves calculated from MLR-predicted PBPK model parameters with those obtained from the MCMC-fitted PBPK model parameters.

To assess the significance of each nanoparticle property in predicting biodistribution behavior, we evaluated its frequency of inclusion in the best subset of multivariate linear regression (MLR) model equations for predicting kinetic metrics and PBPK model parameters. This process involved counting how often each property appeared in the final selected best MLR models. Properties were then ranked based on their frequency of occurrence, producing an ordered list highlighting those with the greatest to least influence on the predictive performance of the MLR model.

## Supplementary Material



## Data Availability

All data and computational models generated during this research are accessible on GitHub: https://github.com/jimengwu/Mouse-general-PBPK.git.

## Notes

Detailed descriptions of the probability distributions used in the Bayesian MCMC model; equations describing nanoparticle concentration kinetics in organs; overall structure of the PBPK model (Figure S1); kinetic indicator diagram (Figure S2); global evaluation of the goodness of model fit with time labeled (Figure S3); goodness of model fit for each experiment (Figure S4); comparison of observed biodistribution data (Figure S5); PBPK model parameter distribution among all nanoparticle experiments (Figure S6); accuracy of biodistribution predictions (Figure S7); evaluation of the predicted kinetic indicators generated from the PBPK MLR model (Figure S8); heat map plot of the nanoparticle properties (Figure S9); overview of chosen properties and their range (Table S1); summary of physiological parameters (Table S2); initial value for kinetic-related parameters (Table S3); assessment of the convergence of MCMC simulations for each experiment (Table S4); accuracy of the MCMC-fitted PBPK model for each nanoparticle experiment (Table S5); summary of multivariable linear regression analysis for predicting PBPK model parameters (Table S6); accuracy of the generated biodistribution curve (Table S7); and summary of multivariable linear regression analysis for predicting kinetic indicators based on nanoparticle properties (Table S8) (PDF)
