# Supplementary material for: Data-Driven Prediction of Nanoparticle Biodistribution from Physicochemical Descriptors
Source: ACS Nano. 2025 Jul 16;19(29):26425–37. doi: 10.1021/acsnano.5c03040 (PMC12312149; doi:10.1021/acsnano.5c03040)
Supplement: Supplementary file 1 [file nn5c03040_si_001.pdf]

Data-Driven Prediction of Nanoparticle Biodistribution from Physicochemical Descriptors

Jimeng Wu[a,b], Peter Wick[a] and Bernd Nowack[b]

[a] Empa, Swiss Federal Laboratories for Materials Science and Technology, Nanomaterials in Health Laboratory, Lerchenfeldstrasse 5, 9014 St. Gallen, Switzerland

[b] Empa, Swiss Federal Laboratories for Materials Science and Technology, Technology and Society Laboratory, Lerchenfeldstrasse 5, 9014 St. Gallen, Switzerland

corresponding author: bernd.nowack@empa.ch

Supporting Information

Table of Contents

1. Supplementary Methods 3

1.1 Probability distribution for Bayesian MCMC model 3

1.2 Equations for Nanoparticle Rate of Change in organs 3

2. Supplementary Tables 7

4. Supplementary Figures 26

1. Supplementary Methods

1.1 Probability distribution for Bayesian MCMC model

The joint posterior distribution of the model parameters (θ) is derived from Bayes' theorem, which states that the posterior distribution is proportional to the product of the likelihood of the observed data and the prior distribution of the parameters. In this case, the predicted nanoparticle (nanoparticle) concentration is modeled as a PBPK model function f of the measured time points t, with a set of parameters θ and an independent variance σ^2, associated with the observed data y. These parameters (θ) are assumed to follow a distribution characterized by the mean μ and variance Σ^2 with the joint probability distribution of θ given by $p(\theta|\mu,\Sigma^2)$. This was specified by a truncated normal model: $log(\theta i) \sim N(\mu,\Sigma^2)$. The hyperprior distributions for μ and Σ^2 are defined as $\log(\mu) \sim N(M,S^2)$, while the variances Σ^2 follow an inverse Gamma distribution $\Gamma^{-1}(3,2\Sigma_0^2)$.

The PBPK model function f links the predicted concentration value to the data (y) and unknown error (ϵ), where the error ϵ is assumed to be log-normally distributed, independent, with a mean of zero and variance σ^2. The prior distribution for σ^2 is log-uniform, with bounds of 0.01 and 3.3.

1.2 Equations for Nanoparticle Rate of Change in organs

A. Blood section

rate of amount nanoparticle changes in venous blood part (mg/ h)

$$\mathrm{RBV} = \mathrm{Q_{Liver}} \times \frac{\mathrm{M_{Liver_b}}}{\mathrm{W_{Liver_b}}} + \mathrm{Q_{Brain}} \times \frac{\mathrm{M_{Brain_b}}}{\mathrm{W_{Brain_b}}} + \mathrm{Q_{Kidney}} \times \frac{\mathrm{M_{Kidney_b}}}{\mathrm{W_{Kidney_b}}} + \mathrm{Q_{Rest}} \times \frac{\mathrm{M_{Rest_b}}}{\mathrm{W_{Rest_b}}} - \mathrm{QC} \times \frac{\mathrm{MBV}}{\mathrm{W_{Blood}} \times 0.8} \qquad [1]$$

rate of change in arterial blood part

$$\mathrm{RBA} = \mathrm{QC} \times \frac{\mathrm{M_{Lung_b}}}{\mathrm{W_{Lung_b}}} - \mathrm{QC} \times \frac{\mathrm{MBA}}{\mathrm{W_{Blood}} \times 0.2} \qquad [2]$$

where MBV is the amount of nanoparticle in venous blood part, MBA is the amount of nanoparticle in arterial blood part, and $\mathrm{W_{Blood}}$ is the weight of blood, QC is the total blood flow, and $\mathrm{Q_{organ}}$ is the blood flow to specific organ, $\mathrm{W_{Organ_b}}$ is the weight of blood inside specific organ.

B. 2 compartment organ

For the 2 compartment organs (brain and remaining tissues), we described the process using the following equations:

rate of concentration changing in organ sub compartment blood compartment:

$$R_o^b = Q_o \times \left(\frac{MBA}{W_{Blood} \times 0.2} - C_o^b\right) - DLC_o \times C_o^b + \frac{DLC_o \times Q_o \times C_o^t}{P_o} \quad [3]$$

rate of concentration changing in organ sub compartment tissue compartment:

$$R_o^t = DLC_o \times C_o^b - \frac{DLC_o \times Q_o \times C_o^t}{P_o} \quad [4]$$

where: Q_o is the blood flow to specific organ, C_o^b, C_o^t, C_o^{pc} is the concentration of nanoparticle in organ blood, tissue, and phagocytic cells, refers to the amount of nanoparticle divided by the weight of compartment respectively, DLC_o is the permeability coefficient between capillary blood and tissue (unitless), P_o is the tissue-to-blood distribution coefficient for organ o (unitless).

C. 3 compartment organ

For the 3 compartment organs (lung, liver, spleen, kidney, gastrointestinal (GI)), without exclusion mentioned below, the nanoparticle rate of change in different organs of the body is described by the following equations:

rate of concentration changing in organ sub compartment blood compartment:

$$R_o^b = Q_o \times (C_B - C_o^b) - DLC_o \times C_o^b + \frac{DLC_o \times Q_o \times C_o^t}{P_o} \quad [5]$$

rate of concentration changing in organ sub compartment tissue compartment:

$$R_o^t = DLC_o \times C_o^b - \frac{DLC_o \times Q_o \times C_o^t}{P_o} - R_o^{uptake} + R_o^{release} \quad [6]$$

rate of concentration changing in organ sub compartment phagocytic cell compartment:

$$R_o^{pc} = R_o^{uptake} - R_o^{release} = K_o^{uptake} \times M_o^t - K_o^{release} \times M_o^{pc} \quad [7]$$

Where C_B refers to concentration in the inflow blood to specific organ, whereas for lung is $\frac{MBV}{W_{Blood} \times 0.8}$ and for others are $\frac{MBA}{W_{Blood} \times 0.2}$, K_o^{uptake} is the uptake rate constant by endocytic or phagocytic cells (1/h), $K_o^{release}$ is the exocytic release rate of nanoparticles from endocytic or phagocytic cells (1/h).

D. Specific organs

Liver-Specific Equations:

In the liver, the phagocytic cell compartment is designed within the blood, and it will have excretion process. The equations for the liver are:

rate of concentration changing inside liver - blood compartment:

$$\begin{aligned} R^{b}_{Liver} = Q_{Liver} \times (C_{B} - C^{b}_{Liver}) + Q_{GI} \times C^{b}_{GI} + Q_{Spleen} \times C^{b}_{Spleen} \\ - DLC_{Liver} \times C^{b}_{Liver} + \frac{DLC_{Liver} \times Q_{Liver} \times C^{t}_{Liver}}{P_{Liver}} \\ - K^{uptake}_{Liver} \times (1 - \frac{M^{pc}_{Liver}}{A^{cap}_{Liver} \times W^{t}_{Liver}}) \times M^{b}_{Liver} + K^{release}_{Liver} \times M^{pc}_{Liver} \end{aligned} \quad [8]$$

rate of concentration changing inside liver - tissue compartment:

$$R^{t}_{Liver} = DLC_{Liver} \times C^{b}_{Liver} - \frac{DLC_{Liver} \times Q_{Liver} \times C^{t}_{Liver}}{P_{Liver}} - K_{bile} \times C^{t}_{Liver} \quad [9]$$

rate of concentration changing inside liver - Phagocytic cell compartment:

$$\begin{aligned} R^{pc}_{Liver} = R^{uptake}_{Liver} - R^{release}_{Liver} \\ = K^{uptake}_{Liver} \times (1 - \frac{M^{pc}_{Liver}}{A^{cap}_{Liver} \times W^{t}_{Liver}}) \times M^{b}_{Liver} - K^{release}_{Liver} \times M^{pc}_{Liver} \end{aligned} \quad [10]$$

Spleen-Specific Equations:

In the spleen, the phagocytic (PC) compartment is designed within the blood. The equations for the spleen are:

rate of concentration changing inside spleen - blood compartment:

$$\begin{aligned} R^{b}_{Spleen} = Q_{Spleen} \times (C_{B} - C^{b}_{Spleen}) - DLC_{Spleen} \times C^{b}_{Spleen} \\ + \frac{DLC_{Spleen} \times Q_{Spleen} \times C^{t}_{Spleen}}{P_{Spleen}} - K^{uptake}_{Spleen} \times M^{b}_{Spleen} \\ + K^{release}_{Spleen} \times M^{pc}_{Spleen} \end{aligned} \quad [11]$$

rate of concentration changing inside spleen - tissue compartment:

$$R^{t}_{Spleen} = DLC_{Spleen} \times C^{b}_{Spleen} - \frac{DLC_{Spleen} \times Q_{Spleen} \times C^{t}_{Spleen}}{P_{Spleen}} \quad [12]$$

rate of concentration changing inside spleen - Phagocytic compartment:

$$R^{pc}_{Spleen} = R^{uptake}_{Spleen} - R^{release}_{Spleen} = K^{uptake}_{Spleen} \times M^{b}_{Spleen} - K^{release}_{Spleen} \times M^{pc}_{Spleen} \quad [13]$$

Kidney-Specific Equation:

The kidney has excretion process which was put in the blood compartment, the tissue and pc compartment stays the same as stated in the 3-compartment organ section. The specific equations for the kidney are:

rate of concentration changing inside kidney - blood compartment:

$$\begin{aligned} R^{b}_{Kidney} &= Q_{Kidney} \times \left(C_{B} - C^{b}_{Kidney}\right) - DLC_{Kidney} \times C^{b}_{Kidney} \\ &+ \frac{DLC_{Kidney} \times Q_{Kidney} \times C^{t}_{Kidney}}{P_{Kidney}} - K_{urine} \times C^{b}_{Kidney} \end{aligned} \qquad [14]$$

GI-Specific Equations:

The tissue and pc compartment stays the same as stated in the 3-compartment organ section. The specific equations for the GI are:

rate of concentration changing inside GI - blood compartment:

$$R^{b}_{GI} = Q_{GI} \times \left(C_{B} - C^{b}_{GI}\right) - DLC_{GI} \times C^{b}_{GI} + \frac{DLC_{GI} \times Q_{GI} \times C^{t}_{GI}}{P_{GI}} + K^{b}_{GI} \times M^{GI_{lumen}} \qquad [15]$$

rate of concentration changing inside GI - lumen compartment

$$R_{GI_{lumen}} = -K^{b}_{GI} \times M^{GI_{lumen}} + K_{bile} \times C^{t}_{Liver} - K_{fecal} \times M^{GI_{lumen}} \qquad [16]$$

2. Supplementary Tables

Table S1. Overview of chosen properties and their range, including core material, coating, shape, hydrodynamic diameter, zeta potential category, and injected dose.

**property**	**unit**	**range**
Core material	-	Iron oxide, silica, Au, GO, TiO_2
coating	-	Ethylenediaminetetraacetic acid (EDTA), dextran, amino groups, polyethylene glycol (PEG), hydrated amorphous silica
Shape of materials	-	Spherical, sheet, rod
Hydrodynamic diameter	nm	[29, 914]
Zeta potential category	-	Negative, positive, neutral, no information
Injected dose	mg/kg	[0.85, 60.4]

Table S2. Summary of physiological parameters baseline value for mousse PBPK model

**Parameter**	**Definition**	**Units**	**Baseline value**	**Source**
BW	Body weight	kg	0.02	[1]
F_{Liver}^{W}	Fractional liver weight	-	0.055	[2]
F_{Brain}^{W}	Fractional brain weight	-	0.017	[2]
F_{Lung}^{W}	Fractional lung weight	-	0.007	[2]
F_{Kidney}^{W}	Fractional kidney weight	-	0.017	[2]
F_{Spleen}^{W}	Fractional spleen weight	-	0.005	[2]
F_{GI}^{W}	Fractional GI tract weight	-	0.0422	[2]
F_{Blood}^{W}	Fractional blood weight	-	0.049	[2]
QCC	Cardiac output	L/h	0.8388	[2]
F_{Brain}^{Q}	Fractional blood flow to brain	-	0.033	[2]
F_{Liver}^{Q}	Fractional blood flow to liver	-	0.161	[2]
F_{Kidney}^{Q}	Fractional blood flow to kidney	-	0.091	[2]
F_{Spleen}^{Q}	Fractional blood flow to spleen	-	0.011	[1]
F_{GI}^{Q}	Fractional blood flow to GI	-	0.215	[1]
F_{Liver}^{BV}	Fractional blood volume in liver	-	0.31	[2]

F_{Brain}^{BV}	Fractional blood volume in brain	-	0.03	[2]
F_{Kidney}^{BV}	Fractional blood volume in kidney	-	0.24	[2]
F_{Spleen}^{BV}	Fractional blood volume in spleen	-	0.17	[2]
F_{Lung}^{BV}	Fractional blood volume in lung	-	0.5	[2]
F_{GI}^{BV}	Fractional blood volume in GI	-	0.04	[2,3]
F_{Rest}^{BV}	Fractional blood volume in rest of body	-	0.04	[2]

Table S3. Initial value for kinetic related parameters inside mouse PBPK model

**Parameter**	**Definition**	**Units**	**Value**
P_{Liver}	Tissue: blood partition coefficient between liver and blood	-	0.08
P_{Brain}	Tissue: blood partition coefficient between brain and blood	-	0.15
P_{Kidney}	Tissue: blood partition coefficient between kidney and blood	-	0.15
P_{Spleen}	Tissue: blood partition coefficient between spleen and blood	-	0.15
P_{Lung}	Tissue: blood partition coefficient between lung and blood	-	0.15
P_{GI}	Tissue: blood partition coefficient between GI and blood	-	0.15
P_{Rest}	Tissue: blood partition coefficient between rest of body and blood	-	0.15
DLC_{Liver}	Permeability coefficient between capillary blood and liver	-	0.001
DLC_{Brain}	Permeability coefficient between capillary blood and brain	-	1.00E-06
DLC_{Kidney}	Permeability coefficient between capillary blood and kidney	-	0.001
DLC_{Spleen}	Permeability coefficient between capillary blood and spleen	-	0.03
DLC_{Lung}	Permeability coefficient between capillary blood and lung	-	0.001
DLC_{GI}	Permeability coefficient between capillary blood and GI	-	0.001
DLC_{Rest}	Permeability coefficient between capillary blood and rest of body	-	1.00E-06
$K_{Liver}^{release}$	Release rate from phagocytic cells to liver	h-1	0.001
$K_{GI}^{release}$	Release rate from phagocytic cells to GI	h-1	0.001
$K_{Spleen}^{release}$	Release rate from phagocytic cells to spleen	h-1	0.001
$K_{Kidney}^{release}$	Release rate from phagocytic cells to kidney	h-1	0.0004
$K_{lung}^{release}$	Release rate from phagocytic cells to lung	h-1	0.003

K_{Liver}^{uptake}	Uptake rate from liver to phagocytic cells	h-1	20
K_{GI}^{uptake}	Uptake rate from GI to phagocytic cells	h-1	0.075
K_{Spleen}^{uptake}	Uptake rate from spleen to phagocytic cells	h-1	40
K_{Kidney}^{uptake}	Uptake rate from kidney to phagocytic cells	h-1	0.075
K_{Lung}^{uptake}	Uptake rate from lung to phagocytic cells	h-1	0.075
K_{bile}	Biliary excretion rate constant	L/h	3.00E-05
K_{urine}	urinary excretion rate constant	L/h	3.00E-06
K_{fecal}	feci excretion rate constant	L/h	3.00E-06
A_{Liver}^{cap}	Uptake capacity per tissue weight	ug/kg tissue	1000
K_{GI}^{b}	absorption rate of GI tract	h-1	4.00E-05

Table S4. Assessment of the convergence of Markov Chain Monte Carlo (MCMC) simulations for each experiment using R-hat statistics, MPSRF is a multivariate generalization of the R-hat statistic, assessing convergence across all parameters simultaneously.

**Experiment ID**	**Experiment name**	**MPSRF**
1	Iron_Oxide_29nm_5mg/kg	1.089
2	Iron_Oxide_41nm_4mg/kg	1.129
3	SiO_2_20nm_10mg/kg	1.111
4	SiO_2_80nm_10mg/kg	1.057
5	Au_12nm_0.85mg/kg	1.089
6	Au_23nm_0.85mg/kg	1.064
7	Au_100nm_0.85mg/kg	1.079
8	Au_34.6nm_3mg/kg	1.072
9	Au_55.5nm_3mg/kg	1.063
10	Au_77.1nm_3mg/kg	1.050
11	Au_82.6nm_3mg/kg	1.061
12	Au_27.6nm_0.85mg/kg	1.043
13	Au_27.6nm_4.26mg/kg	1.059
14	GO_20nm_20mg/kg	1.104
15	GO_243nm_1mg/kg	1.067
16	GO_914nm_1mg/kg	1.062
17	TiO2_385nm_10mg/kg	1.112
18	TiO2_220nm_60mg/kg	1.101

Table S5. Accuracy of MCMC fitted-PBPK Model for each nanoparticle experiment, compared against the observed time-concentration data points.

Experiment ID	Experiment name	$Adj\ R^2$	NRMSE
1	Iron_Oxide_29nm_5mg/kg	0.7	0.6
2	Iron_Oxide_41nm_4mg/kg	0.87	0.72
3	SiO_2_20nm_10mg/kg	0.95	0.21
4	SiO_2_80nm_10mg/kg	0.84	0.39
5	Au_12nm_0.85mg/kg	0.86	0.64
6	Au_23nm_0.85mg/kg	0.89	0.71
7	Au_100nm_0.85mg/kg	0.75	0.45
8	Au_34.6nm_3mg/kg	0.68	0.39
9	Au_55.5nm_3mg/kg	0.87	0.25
10	Au_77.1nm_3mg/kg	0.93	0.28
11	Au_82.6nm_3mg/kg	0.96	0.22
12	Au_27.6nm_0.85mg/kg	0.99	0.43
13	Au_27.6nm_4.26mg/kg	0.99	0.19
14	GO_20nm_20mg/kg	0.48	0.68
15	GO_243nm_1mg/kg	0.94	0.31
16	GO_914nm_1mg/kg	0.98	0.18
17	TiO2_385nm_10mg/kg	0.9	0.33
18	TiO2_220nm_60mg/kg	1	0.1

Table S6. Summary of Multivariable linear Regression Analysis for predicting PBPK model parameters. The table displays the adjusted R^2 values, Akaike Information Criterion (AIC) scores, p value and best multivariable linear regression model of the PBPK model parameters. Predictors are nanoparticle core type (NP), hydrodynamic size (HD), zeta potential (ZP) category, coating (CO), shape (SP), and dose.

**Variable**	$Adj\ R^2$	**AIC**	**P_value**	**Best Equation**
$\log(K_{Liver}^{release})$	0.020	100.125	2.628E-01	$Y = -5.8018 - 0.0704 * Dose$
$\log(K_{Liver}^{uptake})$	0.761	67.414	2.690E-04	$Y = -3.6241 + NP * \begin{pmatrix} GO & 1.9939 \\ Iron\ Oxide & 7.0869 \\ Silica & -0.7034 \\ TiO2 & -0.7423 \end{pmatrix} + 0.069 * Dose$
$\log(A_{Liver}^{cap})$	0.902	30.135	2.029E-04	$Y = 2.0241 + CO * \begin{pmatrix} dextran & -0.6799 \\ EDT & 1.6956 \\ hydrated\ amorphous\ silica & -7.647 \\ PEG & 2.2568 \\ none & 9.8127 \end{pmatrix} + ZP * \begin{pmatrix} neutral & 1.2885 \\ no\ info & 2.0587 \end{pmatrix} + SP * \begin{pmatrix} rod & * \\ sheet & -6.7425 \\ spherical & * \end{pmatrix} + 0.2025 * Dose$

$\log(K_{GI}^{release})$	0.967	-1.164	8.671E-08	$Y = -6.7622 + CO * \begin{pmatrix} dextran & -0.4893 \\ EDT & 0.9517 \\ hydrated\ amorphous\ silica & -4.424 \\ PEG & -0.5134 \\ none & -0.6575 \end{pmatrix} + ZP * \begin{pmatrix} neutral & 0.1821 \\ no\ info & -0.0402 \end{pmatrix}$
$\log(K_{GI}^{uptake})$	0.844	48.549	4.791E-04	$Y = -1.0551 - 0.0039 * HD + CO * \begin{pmatrix} dextran & -0.6188 \\ EDT & -3.5391 \\ hydrated\ amorphous\ silica & 7.169 \\ PEG & -0.6506 \\ none & 1.145 \end{pmatrix} + ZP * \begin{pmatrix} neutral & 0.2601 \\ no\ info & -1.1484 \end{pmatrix}$
$\log(K_{GI}^{b})$	0.927	-17.894	2.531E-07	$Y = -10.5615 + CO * \begin{pmatrix} dextran & 0.0689 \\ EDT & -1.7871 \\ hydrated\ amorphous\ silica & -0.4844 \\ PEG & 0.0579 \\ none & -0.0546 \end{pmatrix}$
$\log(K_{Spleen}^{release})$	0.164	99.492	5.370E-02	$Y = -6.6038 - 0.1241 * Dose$

$\log(K^{uptake}_{Spleen})$	0.666	79.910	1.156E-02	$Y = -7.8581 + 0.0157 * HD + CO * \begin{pmatrix} dextran & -1.7764 \\ EDT & 5.6317 \\ hydrated\ amorphous\ silica & -9.0645 \\ PEG & 0.9886 \\ none & -4.286 \end{pmatrix} + ZP * \begin{pmatrix} neutral & 3.8641 \\ no\ info & 5.0489 \end{pmatrix}$
$\log(K^{release}_{Kidney})$	0.482	69.049	6.405E-03	$Y = -7.9816 + ZP * \begin{pmatrix} neutral & 3.6776 \\ no\ info & 0.8111 \end{pmatrix} - 0.0376 * Dose$
$\log(K^{uptake}_{Kidney})$	0.089	95.267	2.440E-01	$Y = -6.9898 + 0.0043 * HD + ZP * \begin{pmatrix} neutral & 4.6659 \\ no\ info & 1.956 \end{pmatrix}$
$\log(K^{release}_{Lung})$	0.480	89.149	4.530E-02	$Y = 0.7222 + CO * \begin{pmatrix} dextran & -4.4622 \\ EDT & -8.1519 \\ hydrated\ amorphous\ silica & -6.546 \\ PEG & -5.579 \\ none & -4.3547 \end{pmatrix} + ZP * \begin{pmatrix} neutral & 2.0756 \\ no\ info & -2.3985 \end{pmatrix}$
$\log(K^{uptake}_{Lung})$	0.657	81.337	4.087E-03	$Y = -5.7676 + 0.0094 * HD + CO * \begin{pmatrix} dextran & -8.925 \\ EDT & 1.7123 \\ hydrated\ amorphous\ silica & -5.1932 \\ PEG & -6.6225 \\ none & -7.5771 \end{pmatrix} + ZP * \begin{pmatrix} neutral & 10.0674 \\ no\ info & 5.5197 \end{pmatrix}$

$\log(P_{Liver})$	0.066	60.354	2.336E-01	$Y = -2.8861 + ZP * \begin{pmatrix} neutral & 1.2811 \\ no\ \ info & 0.1569 \end{pmatrix}$
$\log(P_{Brain})$	0.874	-23.573	2.158E-05	$Y = -2.2953 - 3e^{-4} * HD + CO * \begin{pmatrix} dextran & -0.0962 \\ EDT & 0.616 \\ hydrated\ \ amorphous\ \ silica & 1.0772 \\ PEG & 0.016 \\ none & 0.2299 \end{pmatrix}$
$\log(P_{Kidney})$	0.407	72.335	5.760E-02	$Y = -0.4533 + NP * \begin{pmatrix} GO & -1.7395 \\ Iron\ \ Oxide & 0.6479 \\ Silica & -2.666 \\ TiO2 & -3.5242 \end{pmatrix} + ZP * \begin{pmatrix} neutral & -3.1653 \\ no\ \ info & -0.7071 \end{pmatrix}$
$\log(P_{Spleen})$	0.970	24.393	1.278E-09	$Y = -2.099 + CO * \begin{pmatrix} dextran & 3.6142 \\ EDT & 5.884 \\ hydrated\ \ amorphous\ \ silica & -6.1816 \\ PEG & 0.1629 \\ none & -0.8127 \end{pmatrix}$
$\log(P_{Lung})$	0.871	24.567	2.151E-04	$Y = -4.3275 + 0.0017 * HD + CO * \begin{pmatrix} dextran & 2.3756 \\ EDT & 0.7325 \\ hydrated\ \ amorphous\ \ silica & -9.8819 \\ PEG & 1.1139 \\ none & 3.6762 \end{pmatrix} + SP * \begin{pmatrix} rod & * \\ sheet & -1.4277 \\ spherical & 0.878 \end{pmatrix} + 0.1359 * Dose$

$\log(P_{Rest})$	0.823	-28.809	8.304E-04	$Y = -2.5634 + NP * \begin{pmatrix} GO & -0.0301 \\ Iron\ \ Oxide & -0.1826 \\ Silica & -0.1028 \\ TiO2 & 0.5694 \end{pmatrix} - 8e^{-4} * HD + ZP * \begin{pmatrix} neutral & 0.2937 \\ no\ \ info & 0.3634 \end{pmatrix} - 0.0047 * Dose$
$\log(P_{GI})$	0.988	39.275	5.507E-09	$Y = 5.5588 - 0.0191 * HD + CO * \begin{pmatrix} dextran & -0.6986 \\ EDT & 1.7806 \\ hydrated\ \ amorphous\ \ silica & 22.061 \\ PEG & -0.8241 \\ none & 9.8582 \end{pmatrix} + ZP * \begin{pmatrix} neutral & -5.2914 \\ no\ \ info & -6.3297 \end{pmatrix}$
$\log(DLC_{Liver})$	0.965	5.238	3.446E-06	$Y = -5.9511 - 0.0032 * HD + CO * \begin{pmatrix} dextran & -0.1269 \\ EDT & 0.4928 \\ hydrated\ \ amorphous\ \ silica & -0.1784 \\ PEG & 0.1013 \\ none & 4.7616 \end{pmatrix} + ZP * \begin{pmatrix} neutral & -1.3111 \\ no\ \ info & -1.0489 \end{pmatrix} - 0.0142 * Dose$
$\log(DLC_{Brain})$	0.856	-26.630	1.408E-05	$Y = -14.261 + CO * \begin{pmatrix} dextran & -0.0308 \\ EDT & 1.0082 \\ hydrated\ \ amorphous\ \ silica & 0.3828 \\ PEG & 0.0501 \\ none & 0.0797 \end{pmatrix}$

$\log(DLC_{Kidney})$	0.995	0.853	1.506E-09	$Y = -6.4848 - 0.004 * HD + CO * \begin{pmatrix} dextran & 9.7551 \\ EDT & 4.3368 \\ hydrated\ \ amorphous\ \ silica & -7.4551 \\ PEG & 1.1913 \\ none & 7.3091 \end{pmatrix} + ZP * \begin{pmatrix} neutral & -1.8973 \\ no\ \ info & -1.3481 \end{pmatrix} + SP * \begin{pmatrix} rod & * \\ sheet & -1.833 \\ spherical & * \end{pmatrix} - 0.0693 * Dose$
$\log(DLC_{Spleen})$	0.999	-35.076	3.623E-13	$Y = -3.2775 - 0.0016 * HD + CO * \begin{pmatrix} dextran & 0.7818 \\ EDT & 11.9352 \\ hydrated\ \ amorphous\ \ silica & 2.8594 \\ PEG & 0.1288 \\ none & -0.1738 \end{pmatrix} + ZP * \begin{pmatrix} neutral & -0.6443 \\ no\ \ info & -0.489 \end{pmatrix} - 0.0098 * Dose$
$\log(DLC_{Lung})$	0.986	6.654	1.018E-07	$Y = -4.1313 - 0.0082 * HD + CO * \begin{pmatrix} dextran & 1.6793 \\ EDT & 1.813 \\ hydrated\ \ amorphous\ \ silica & -1.7519 \\ PEG & 0.0881 \\ none & 9.899 \end{pmatrix} + SP * \begin{pmatrix} rod & * \\ sheet & -3.1047 \\ spherical & -2.8033 \end{pmatrix}$

$\log(DLC_{Rest})$	0.289	-24.635	5.176E-02	$Y = -14.026 - 3e^{-4} * HD + ZP * \begin{pmatrix} neutral & -0.1723 \\ no\ \ info & -0.1512 \end{pmatrix}$
$\log(DLC_{GI})$	0.997	2.236	1.352E-10	$Y = -5.0042 - 0.005 * HD + CO * \begin{pmatrix} dextran & -0.4343 \\ EDT & 3.0887 \\ hydrated\ \ amorphous\ \ silica & 17.83 \\ PEG & -0.4082 \\ none & 3.148 \end{pmatrix} + ZP * \begin{pmatrix} neutral & -1.363 \\ no\ \ info & -1.6696 \end{pmatrix} - 0.0137 * Dose$
$\log(K_{bile})$	0.597	88.839	5.090E-03	$Y = -10.843 + CO * \begin{pmatrix} dextran & 12.4566 \\ EDT & -1.3629 \\ hydrated\ \ amorphous\ \ silica & 0.9109 \\ PEG & -1.0492 \\ none & 0.822 \end{pmatrix}$
$\log(K_{urine})$	0.087	109.200	1.972E-01	$Y = -13.6039 + ZP * \begin{pmatrix} neutral & -3.6093 \\ no\ \ info & 1.236 \end{pmatrix}$
$\log(K_{fecal})$	0.800	-23.131	9.520E-05	$Y = -13.1242 + CO * \begin{pmatrix} dextran & 0.048 \\ EDT & -0.8921 \\ hydrated\ \ amorphous\ \ silica & -0.0116 \\ PEG & 0.0241 \\ none & -0.1792 \end{pmatrix}$

Table S7. Accuracy of generated biodistribution curve from Multivariable linear Regression predicted PBPK model parameters compared to observation data points.

**Experiment ID**	**Experiment name**	**Adj R^2**
1	Iron_Oxide_29nm_5mg/kg	0.712
2	Iron_Oxide_41nm_4mg/kg	0.287
3	SiO_2_20nm_10mg/kg	0.414
4	SiO_2_80nm_10mg/kg	0.083
5	Au_12nm_0.85mg/kg	0.730
6	Au_23nm_0.85mg/kg	0.740
7	Au_100nm_0.85mg/kg	0.336
8	Au_34.6nm_3mg/kg	-0.049
9	Au_55.5nm_3mg/kg	0.078
10	Au_77.1nm_3mg/kg	0.324
11	Au_82.6nm_3mg/kg	-0.000
12	Au_27.6nm_0.85mg/kg	0.867
13	Au_27.6nm_4.26mg/kg	0.864
14	GO_20nm_20mg/kg	-0.063
15	GO_243nm_1mg/kg	0.390
16	GO_914nm_1mg/kg	0.939
17	TiO2_385nm_10mg/kg	0.042
18	TiO2_220nm_60mg/kg	0.995

Table S8. Summary of Multivariable linear Regression Analysis for predicting kinetics indicators Based on nanoparticle Properties. The table displays the adjusted R^2 values, Akaike Information Criterion (AIC) scores, p value and best combinations of predictors for each PK parameter. Predictors are nanoparticle core type (NP), hydrodynamic size (HD), zeta potential (ZP) category, coating (CO), shape (SP), and dose.

Variable	$Adj\ R^2$	AIC	p_value	Best Equation
$\log(C_{max}^{Liver})$	0.839	9.129	7.965E-05	$Y = 1.772 - 0.0019 * HD + SP * \begin{pmatrix} rod & * \\ sheet & -0.683 \\ spherical & * \end{pmatrix} - 0.0454 * Dose$
$\log(C_{max}^{Kidney})$	0.769	33.756	2.206E-04	$Y = -0.1733 + CO * \begin{pmatrix} dextran & 1.2122 \\ EDT & 1.7037 \\ hydrated\ amorphous\ silica & -2.7878 \\ PEG & 1.0409 \\ none & 0.3567 \end{pmatrix}$
$\log(C_{max}^{Spleen})$	0.948	21.377	4.102E-06	$Y = 0.0174 + 0.004 * HD + CO * \begin{pmatrix} dextran & -0.1216 \\ EDT & 2.0272 \\ hydrated\ amorphous\ silica & -6.51 \\ PEG & 0.6016 \\ none & -1.9533 \end{pmatrix} + ZP * \begin{pmatrix} neutral & 0.6118 \\ no\ info & 1.2551 \end{pmatrix}$
$\log(C_{max}^{Lung})$	0.958	7.324	1.553E-06	$Y = 0.5967 + 6e-04 * HD +$

				$CO * \begin{pmatrix} dextran & -0.1917 \\ EDT & 0.0439 \\ hydrated\ \ amorphous\ \ silica & -5.3647 \\ PEG & -\ 0.6899 \\ none & -0.5592 \end{pmatrix} +$ $ZP * \begin{pmatrix} neutral & 1.0926 \\ no\ \ info & 0.8222 \end{pmatrix}$
$\log(DE_{24}^{Liver})$	0.740	12.539	2.428E-04	$Y = 1.192 + CO * \begin{pmatrix} dextran & * \\ EDT & * \\ hydrated\ \ amorphous\ \ silica & -1.9875 \\ PEG & 0.1101 \\ none & * \end{pmatrix}$
$\log(DE_{24}^{Kidney})$	0.846	24.648	1.775E-03	$Y = -3.2785 + 0.0083 * HD +$ $CO * \begin{pmatrix} dextran & * \\ EDT & * \\ hydrated\ \ amorphous\ \ silica & -2.8112 \\ PEG & 0.9734 \\ none & * \end{pmatrix} +$ $ZP * \begin{pmatrix} neutral & 2.3438 \\ no\ \ info & 2.9808 \end{pmatrix} - 0.034 * Dose$
$\log(DE_{24}^{Spleen})$	0.969	8.313	2.034E-09	$Y = 1.2966 + CO * \begin{pmatrix} dextran & * \\ EDT & * \\ hydrated\ \ amorphous\ \ silica & -5.7005 \\ PEG & 0.104 \\ none & * \end{pmatrix}$

$\log(DE_{24}^{Lung})$	0.964	6.680	1.200E-05	$Y = 1.6402 - 0.0047 * HD + ZP * \begin{pmatrix} neutral & -9.6914 \\ no\ \ info & -9.9535 \end{pmatrix} + SP * \begin{pmatrix} rod & * \\ sheet & 6.7779 \\ spherical & 8.9908 \end{pmatrix} + 0.0914 * Dose$
$\log(DE_{168}^{Liver})$	0.786	9.729	1.455E-05	$Y = 1.5504 - 0.0369 * Dose$
$\log(DE_{168}^{Kidney})$	0.758	31.780	3.083E-05	$Y = 0.7375 - 0.0751 * Dose$
$\log(DE_{168}^{Spleen})$	0.965	13.501	1.065E-05	$Y = 3.5761 - 0.0043 * HD + CO * \begin{pmatrix} dextran & * \\ EDT & * \\ hydrated\ \ amorphous\ \ silica & -2.798 \\ PEG & 0.0283 \\ none & * \end{pmatrix} + ZP * \begin{pmatrix} neutral & -1.8446 \\ no\ \ info & -1.4934 \end{pmatrix} - 0.0487 * Dose$
$\log(DE_{168}^{Lung})$	0.972	3.627	3.140E-05	$Y = 2.1807 - 0.0068 * HD + ZP * \begin{pmatrix} neutral & -9.7179 \\ no\ \ info & -10.0238 \end{pmatrix} + SP * \begin{pmatrix} rod & * \\ sheet & 5.6855 \\ spherical & 8.3888 \end{pmatrix} + 0.087 * Dose$
$\log(ARA^{Liver})$	0.750	40.013	8.055E-04	$Y = -1.2407 + NP * \begin{pmatrix} GO & 2.2393 \\ Iron\ \ Oxide & 3.1727 \\ Silica & -0.3206 \\ TiO2 & 0.8952 \end{pmatrix} + ZP * \begin{pmatrix} neutral & 1.6683 \\ no\ \ info & 0.6119 \end{pmatrix}$

$\log(ARA^{Kidney})$	0.928	20.392	1.724E-05	$Y = 0.3575 - 0.0018 * HD + CO * \begin{pmatrix} dextran & 3.1969 \\ EDT & 2.5257 \\ hydrated\ amorphous\ silica & -1.4834 \\ PEG & 0.4625 \\ none & 2.5489 \end{pmatrix} + ZP * \begin{pmatrix} neutral & -1.3447 \\ no\ info & -0.3631 \end{pmatrix}$
$\log(ARA^{Spleen})$	0.669	54.093	6.340E-03	$Y = 1.5949 + NP * \begin{pmatrix} GO & 1.3337 \\ Iron\ Oxide & 1.7686 \\ Silica & -0.2595 \\ TiO2 & -2.7752 \end{pmatrix} + 0.0018 * HD + ZP * \begin{pmatrix} neutral & -1.4536 \\ no\ info & -2.1712 \end{pmatrix}$
$\log(ARA^{Lung})$	0.908	29.794	1.531E-04	$Y = -1.4442 + CO * \begin{pmatrix} dextran & 3.779 \\ EDT & 2.7396 \\ hydrated\ amorphous\ silica & -14.6821 \\ PEG & 2.8587 \\ none & 9.3372 \end{pmatrix} + ZP * \begin{pmatrix} neutral & -1.575 \\ no\ info & -0.393 \end{pmatrix} + SP * \begin{pmatrix} rod & * \\ sheet & -4.8768 \\ spherical & * \end{pmatrix} + 0.2283 * Dose$

3. Supplementary Figures

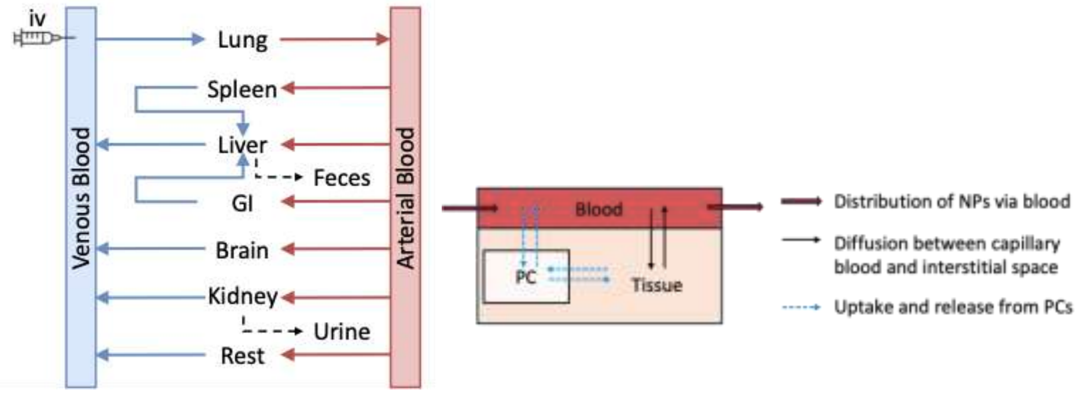

Figure S1. Left: Overall structure of the PBPK model for nanoparticle biodistribution following intravenously administration in the mouse. Right: Organ compartment division. Uptake and release from endocytic or phagocytic cells either from tissue or from blood depends on the mononuclear phagocyte system positions in different organ as shown in the blue dashed arrow.

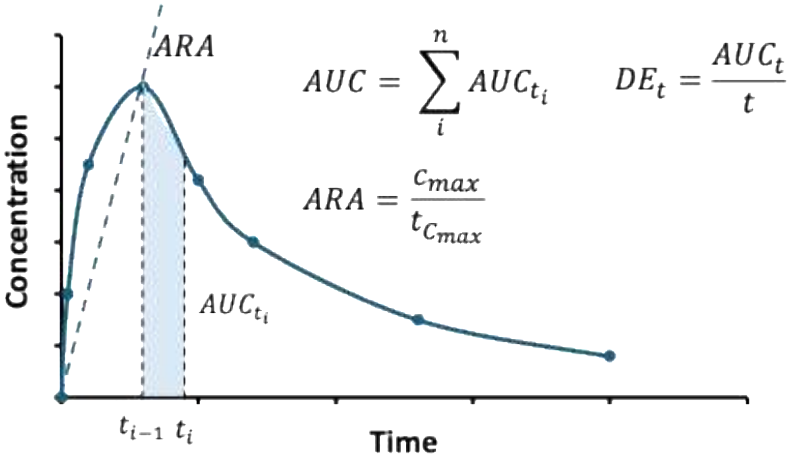

Figure S2. Diagram explaining the Kinetic Indicators. The solid blue dots and line represent the concentration vs. time biodistribution curve for nanoparticles in the organs. The area-under-the-curve (AUC) was calculated using the linear trapezoidal method, capturing the area from t-zero to t-last. Delivery efficiency (DE) was derived by dividing the AUC by the last measured time point, providing an indicator of the nanoparticles' overall persistence within the organ. The average rate of accumulation (ARA) was calculated by dividing the maximum concentration (in ng/g) by the time at which this concentration was reached.

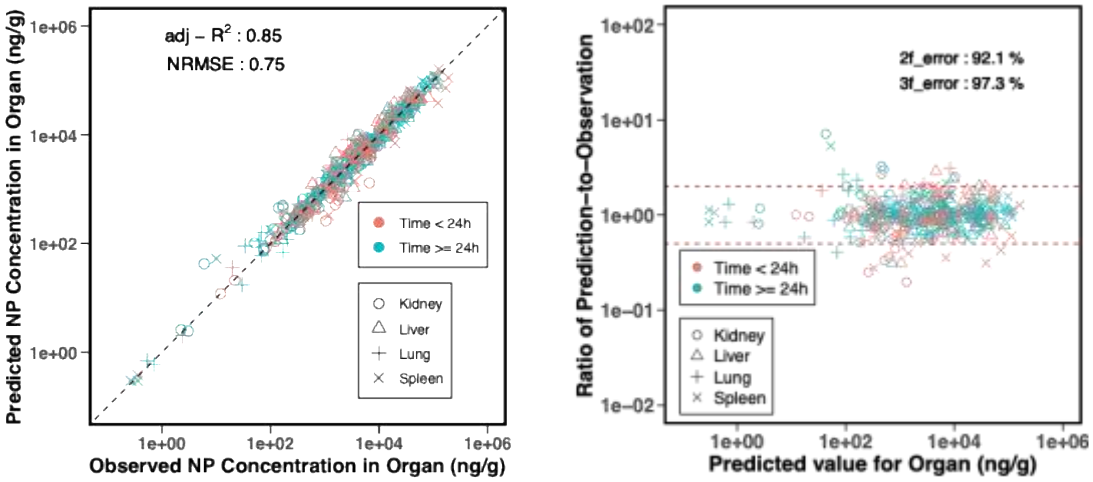

Figure S3. Global evaluation of the goodness of model fit with time labeled. The different symbol shapes are used for different organs, including the kidney (round), liver (triangle), lung (plus), and spleen (cross). In panel left, the solid black diagonal line represents the unity line where the observed and predicted values are equal. The figure is presented on a log-log scale since the errors are assumed to be log-normally distributed and the data span a wider range. In panel right, the dashed line represents a predicted-to-observed ratio of 2 or lower than 0.5.

Experiment 1: Iron_oxide_29nm_5mg/kg

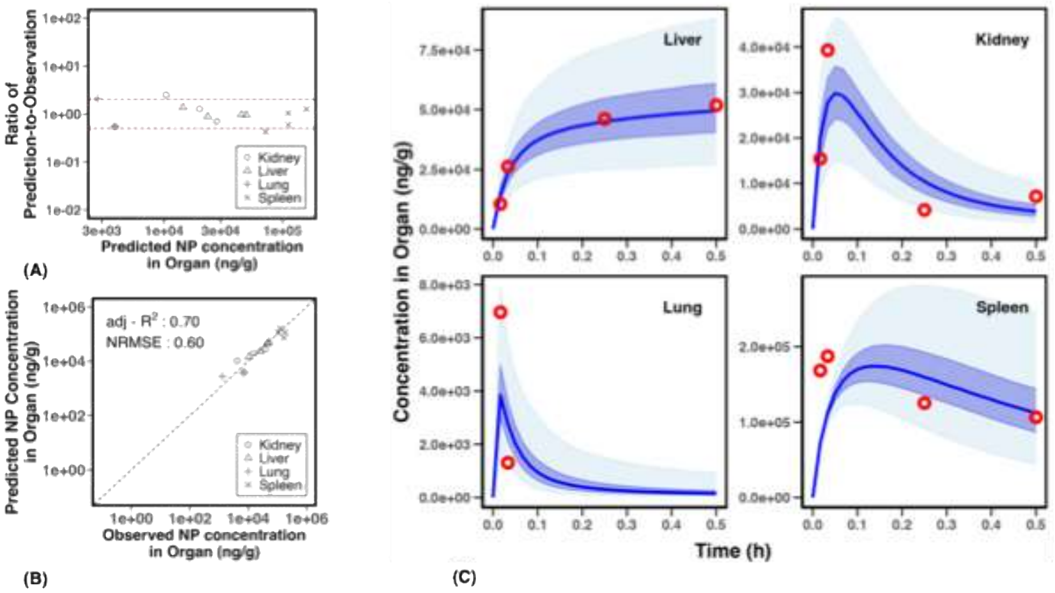

Experiment 2: Iron_oxide_41nm_4mg/kg

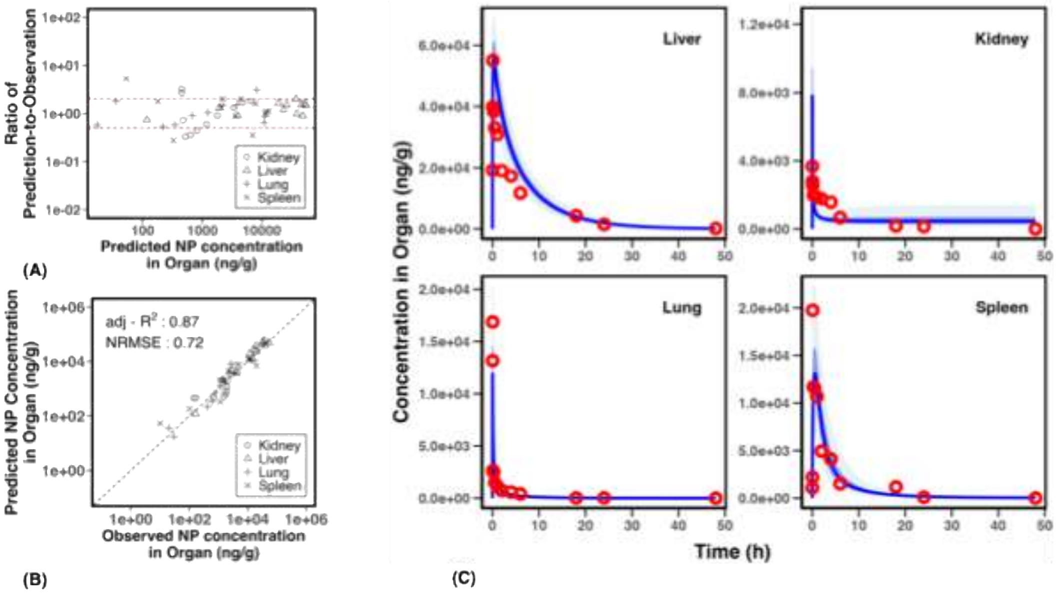

Experiment 3: SiO2_20nm_10mg/kg

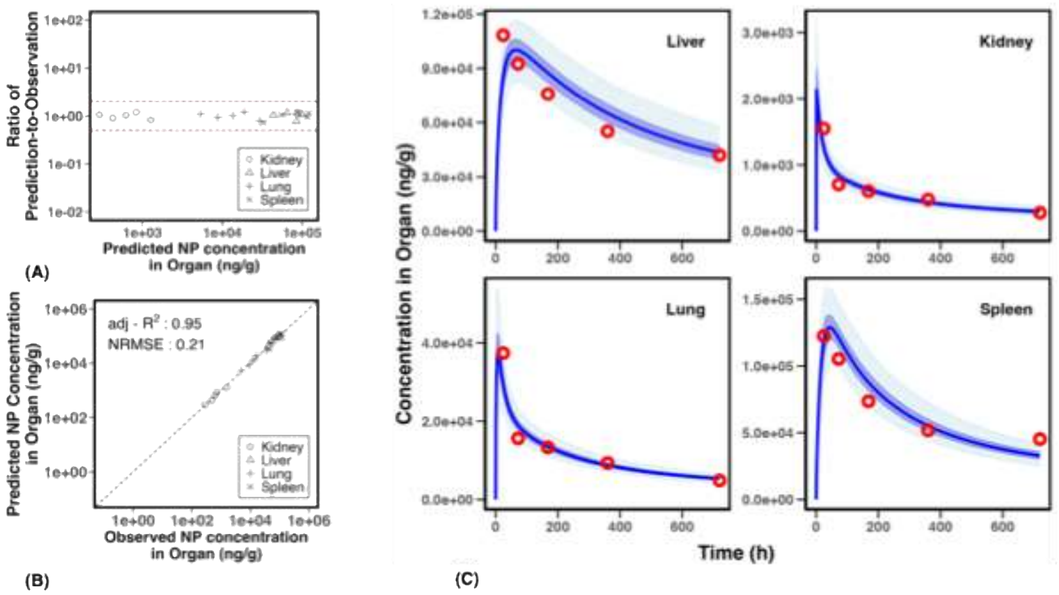

Experiment 4: SiO2_80nm_10mg/kg

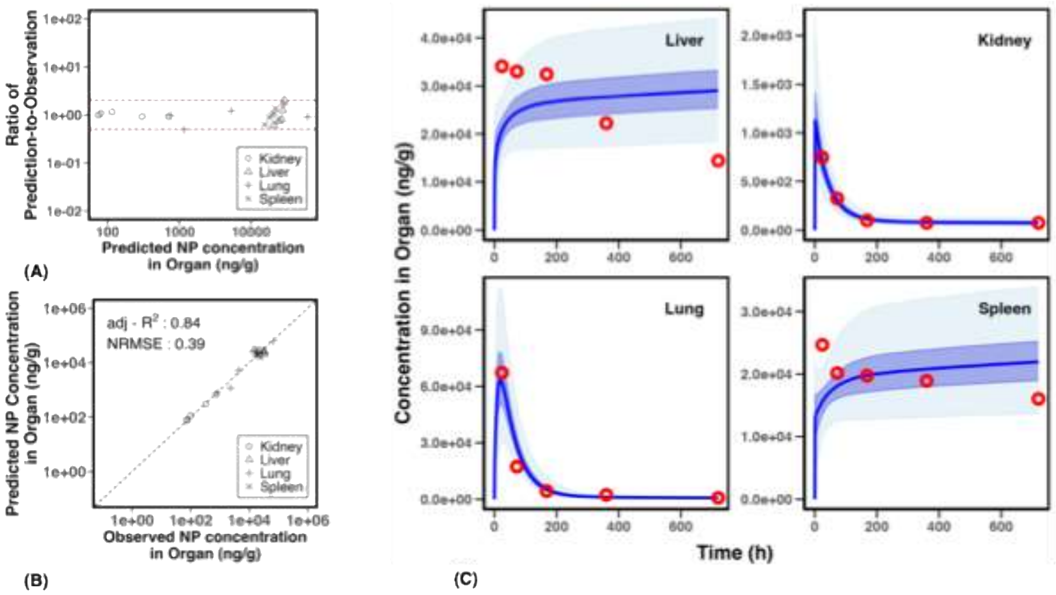

Experiment 5: Au_12nm_0.85mg/kg

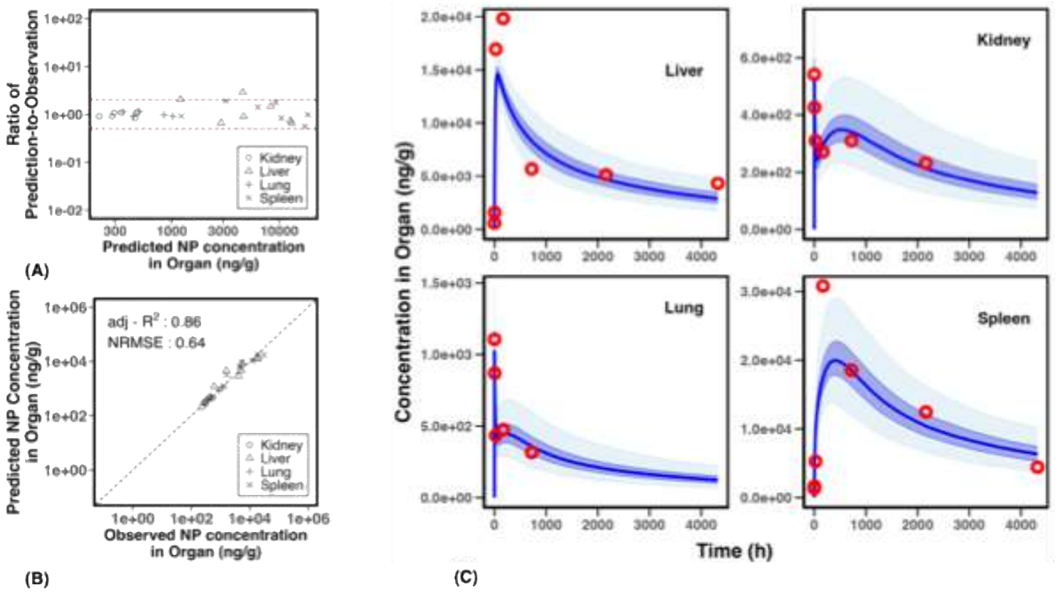

Experiment 6: Au_23nm_0.85mg/kg

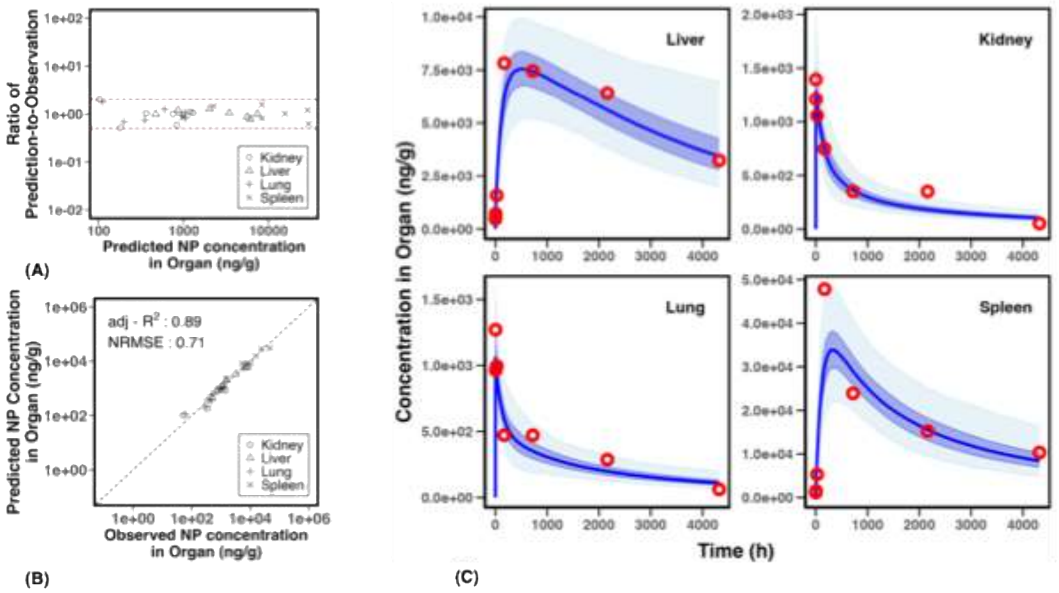

Experiment 7: Au_100nm_0.85mg/kg

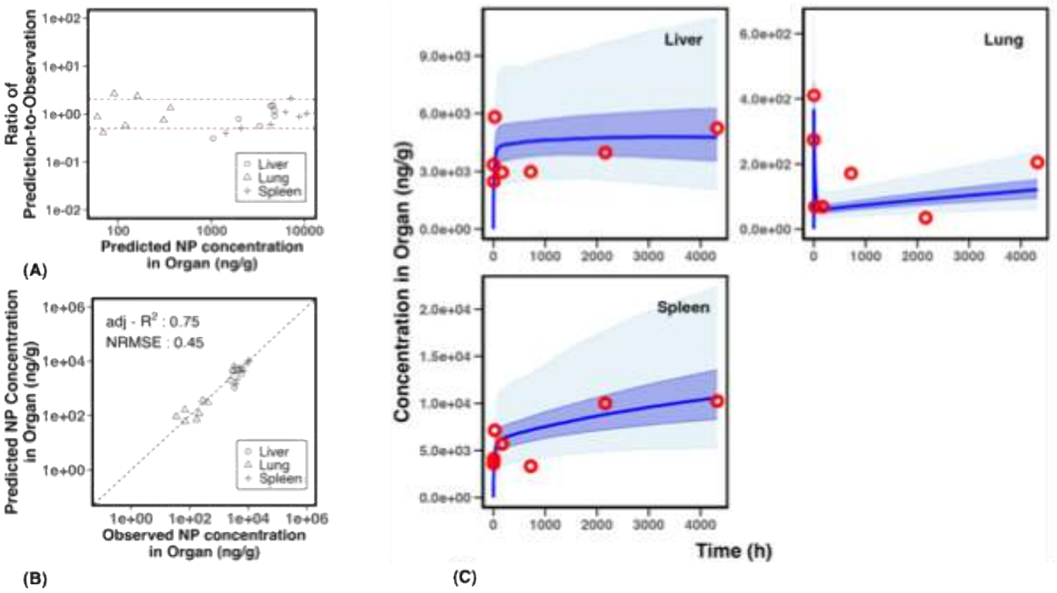

Experiment 8: Au_34.6nm_3mg/kg

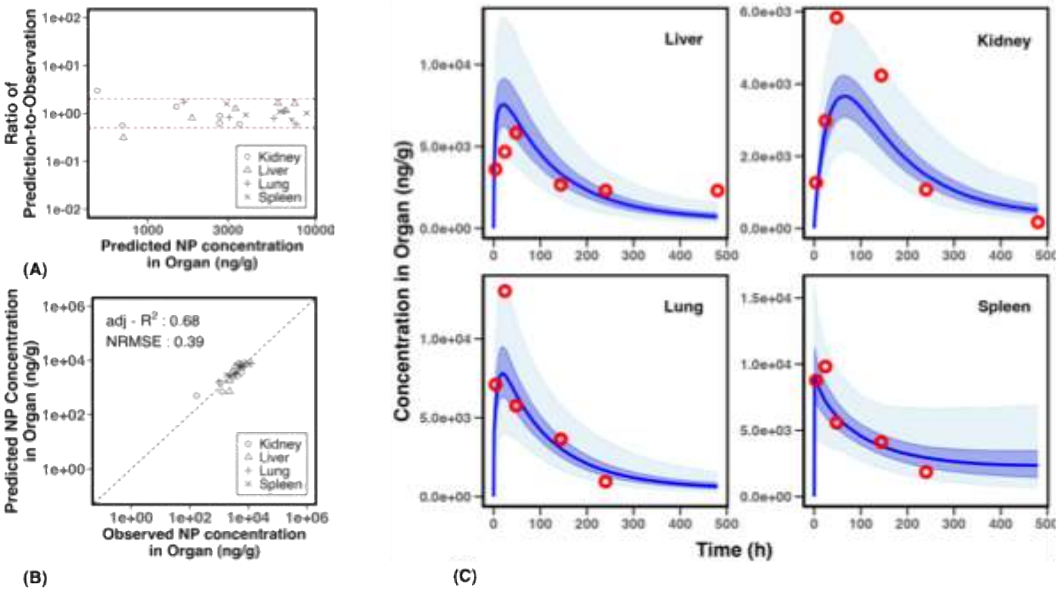

Experiment 9: Au_55.5nm_3mg/kg

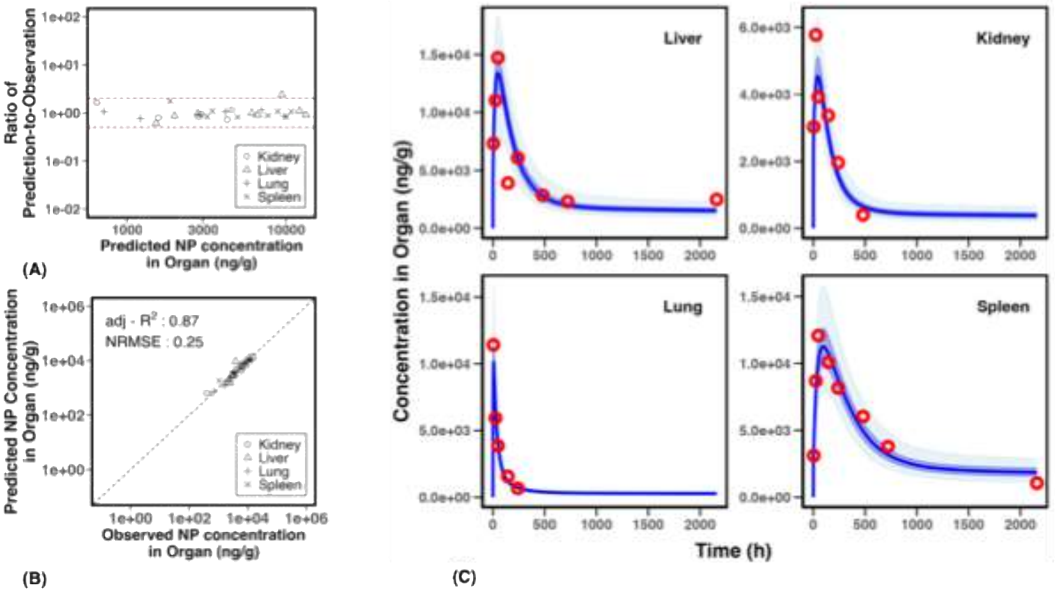

Experiment 10: Au_77.1nm_3mg/kg

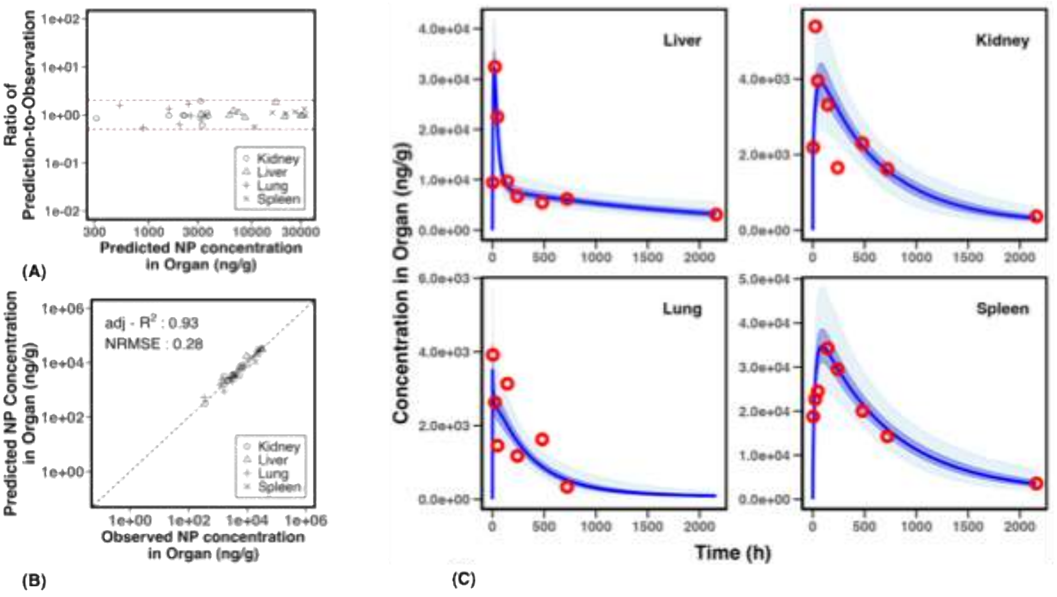

Experiment 11: Au_82.6nm_3mg/kg

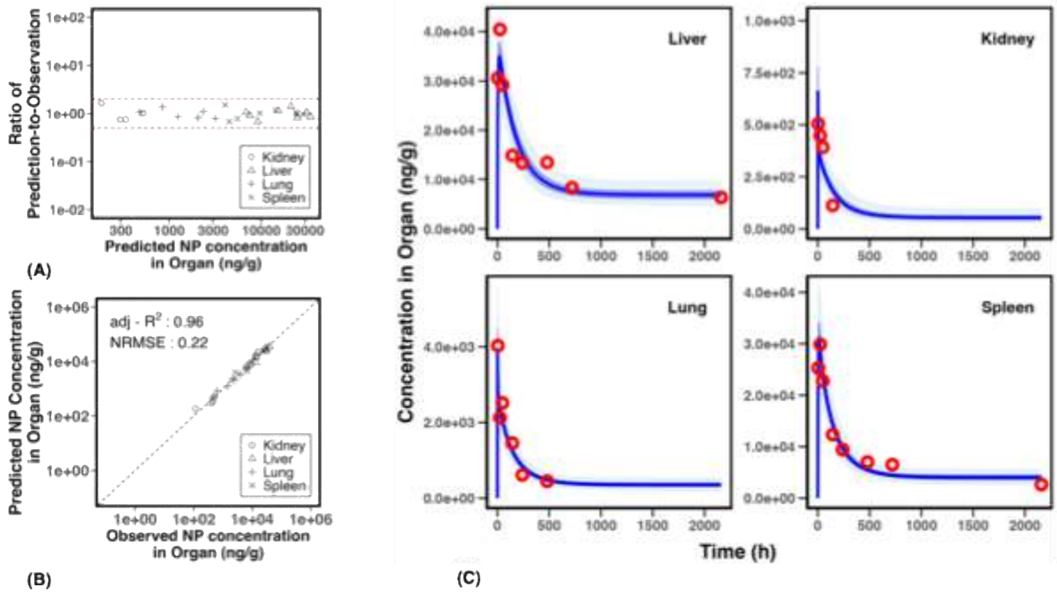

Experiment 12: Au_27.6nm_4.26mg/kg

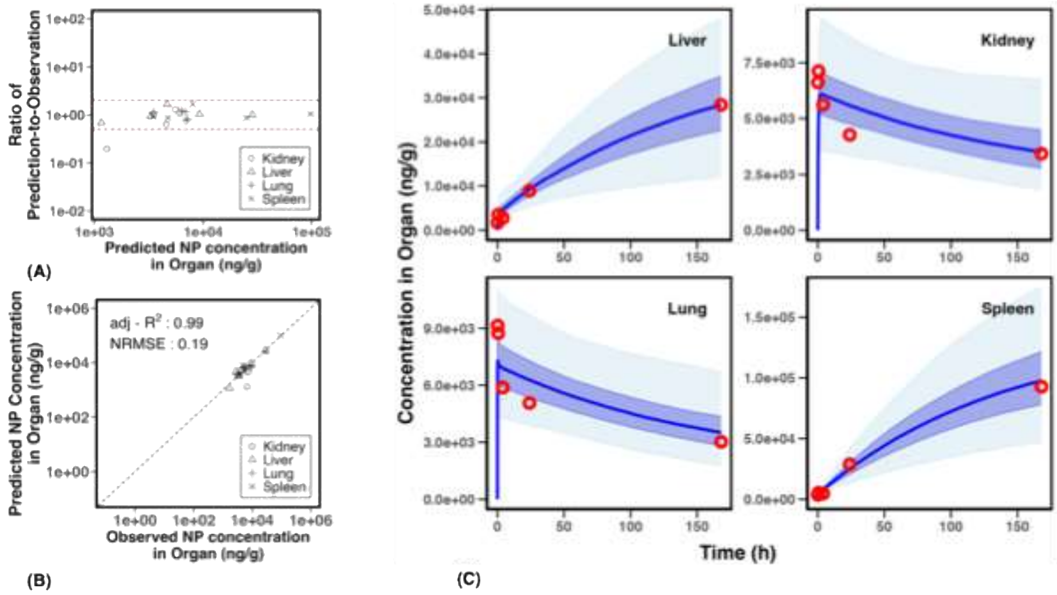

Experiment 13: Au_27.6nm_0.85mg/kg

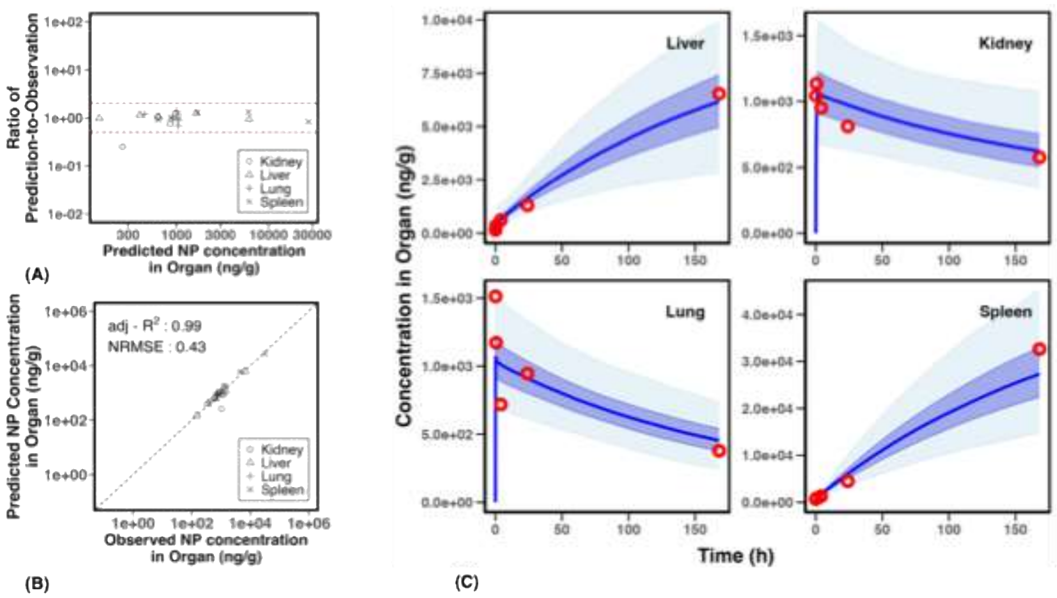

Experiment 14: GO_20nm_20mg/kg

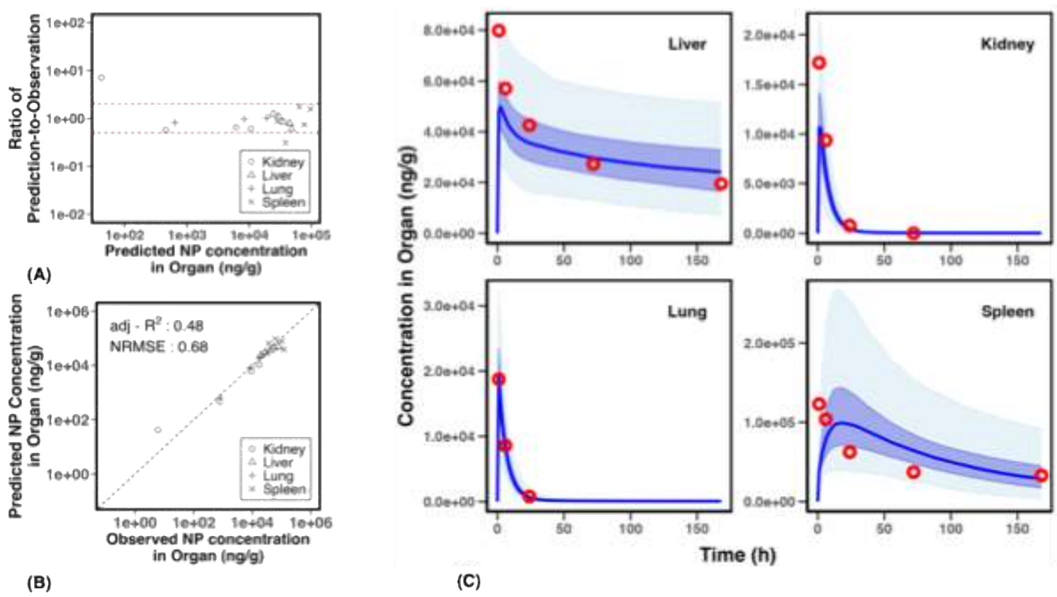

Experiment 15: GO_243nm_1mg/kg

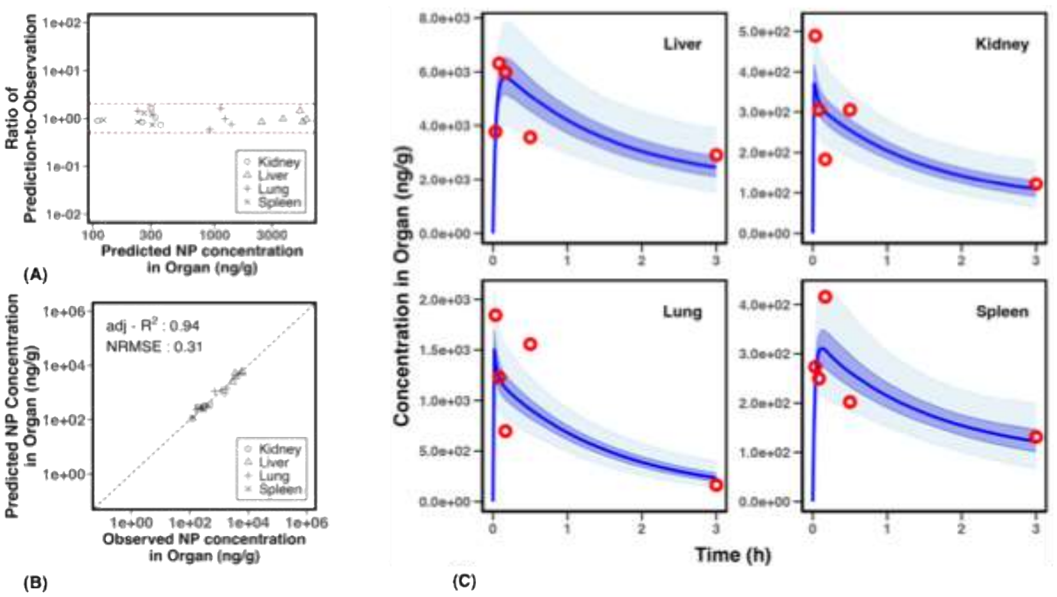

Experiment 16: GO_914nm_1mg/kg_w/o_CS

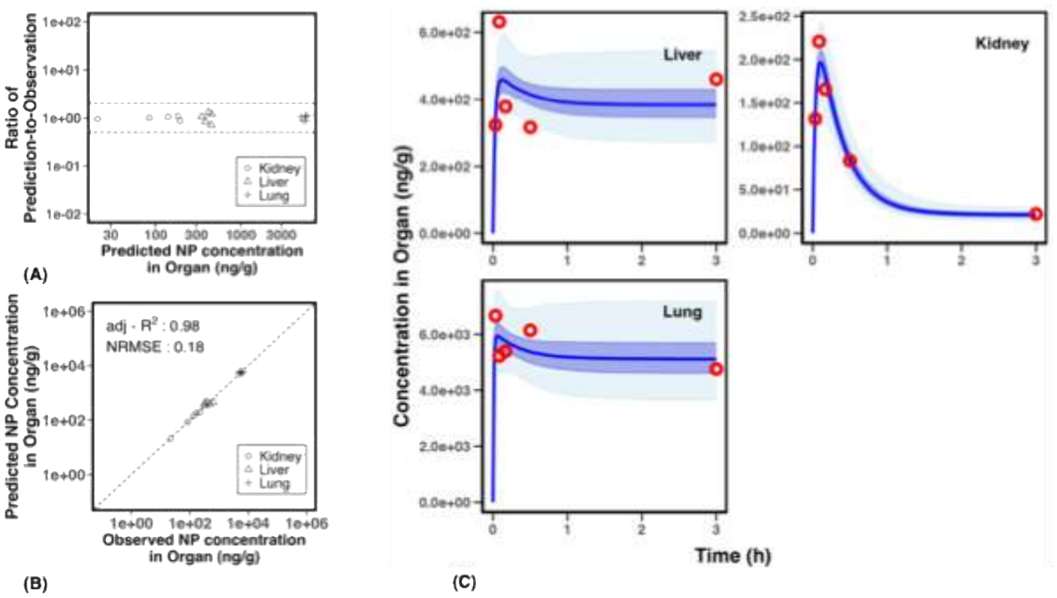

Experiment 17: TiO2_385nm_10mg/kg

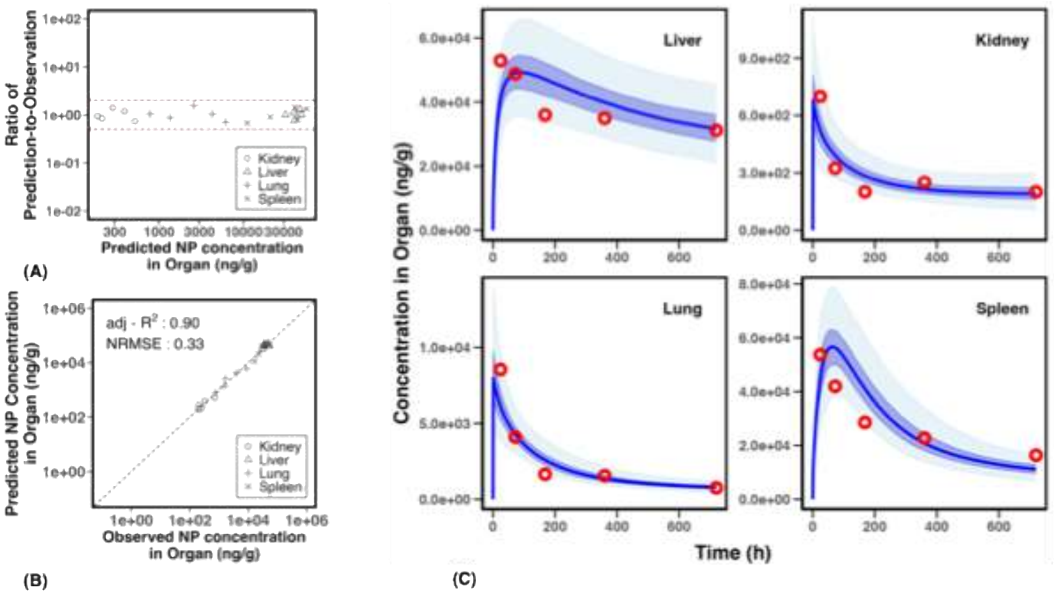

Experiment 18: TiO2_220nm_60mg/kg

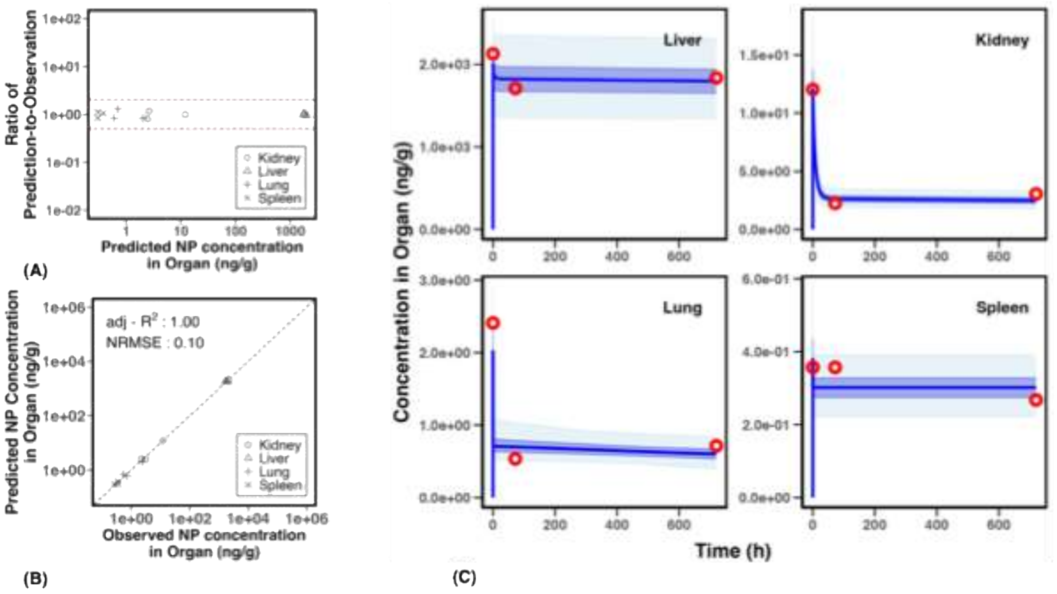

Figure S4. The goodness of model fit for each experiment. The different symbol shapes are used for different organs, including the kidney (round), liver (triangle), lung (plus), and spleen (cross). The panels display: (A) the predicted-to-observed ratio versus model prediction plot, (B) the global evaluation of the PBPK model MCMC fit, and (C) comparisons of the predicted biodistribution curves generated from MCMC-fitted posterior parameters with observed data points. In the first plot, the dashed lines indicate the thresholds for a predicted-to-observed ratio greater than 2 or less than 0.5. In the second plot, the dashed black diagonal line represents the unity line, where

observed and predicted values are equal. The figure is presented on a log-log scale since the errors are assumed to be log-normally distributed and the data span a wider range. In the third plot, the predicted concentration curve was compared with observed data points, the blue curve shows the fitted time-concentration curve, with light and dark shading representing the interquartile range (25%-75%) and the 95% confidence interval, respectively. Red points mark the measured concentrations of nanoparticles in four organs.

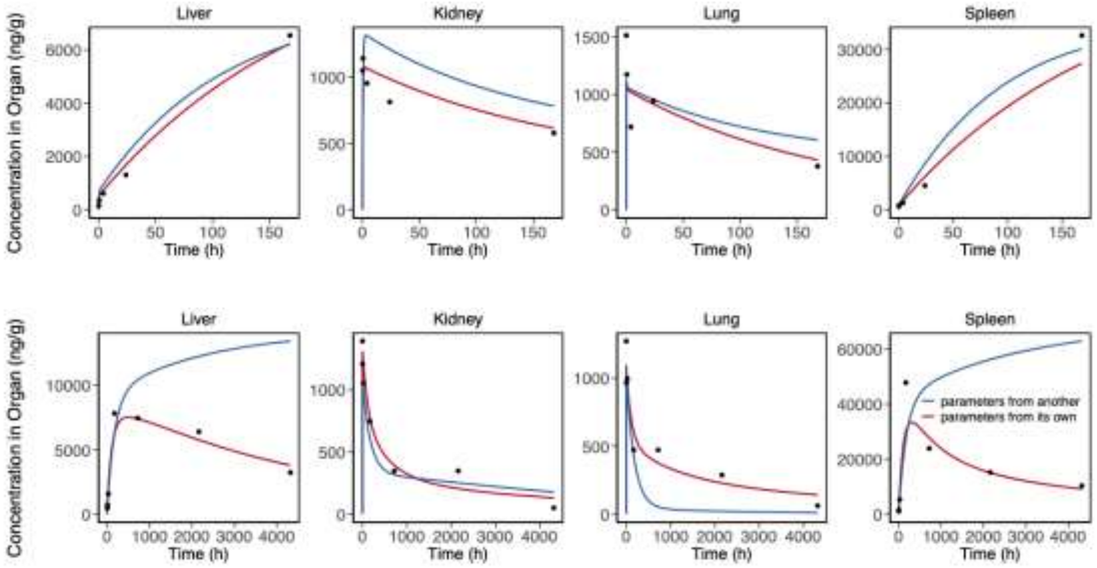

Figure S5. Comparison of observed biodistribution data with PBPK model predictions for two independent nanoparticle experiments under identical conditions but with differing observation periods (one study with 168 h, the other with 4320 h). The black dots represent observed data points. Red lines represent biodistribution curve using parameters obtained from the corresponding experiment, while blue lines show calculation results using the other set of parameters. Top row: Using observation time points from experiment 6 - Au - 23nm - 0.85mg/kg (maximum measurement time 4320h). the red line corresponds to the calculated biodistribution curve using the PBPK model parameters fitted from experiment 6; and blue clue uses the PBPK model parameter fitted from experiment 12. Bottom row: Using observation time points from experiment 12 - Au - 23nm - 0.85mg/kg (maximum measurement time 168h). The red line reflects the biodistribution curve with parameters fitted from experiment 12, while the blue line uses fitted parameters from experiment 6.

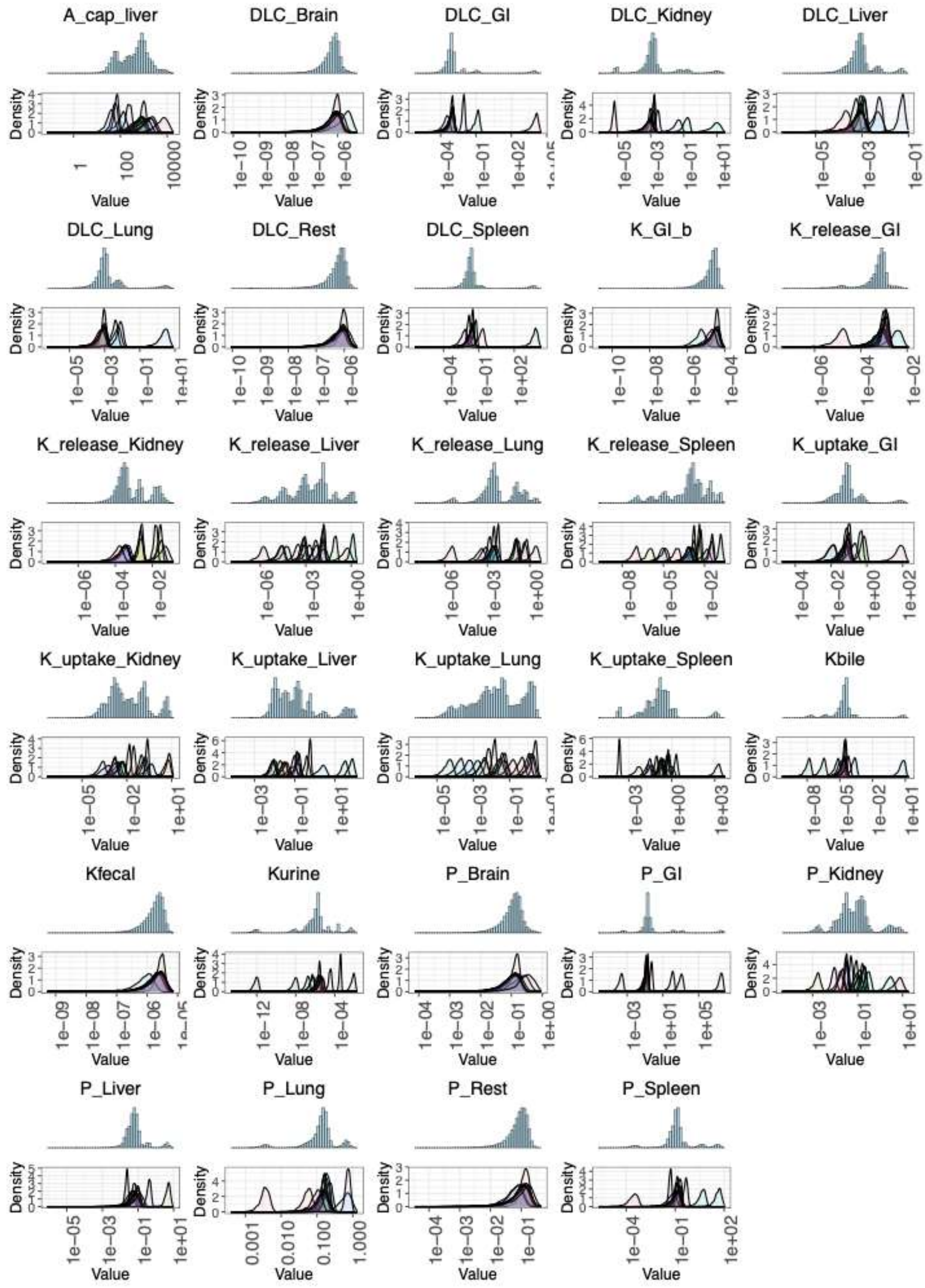

Figure S6. PBPK model parameter distribution among all nanoparticle experiment. Each subplot represents a different PBPK model parameter. The lower part of each subplot displays the overlapping posterior parameter distributions for each nanoparticle study. The upper part represents the frequency of parameter values across all nanoparticle studies.

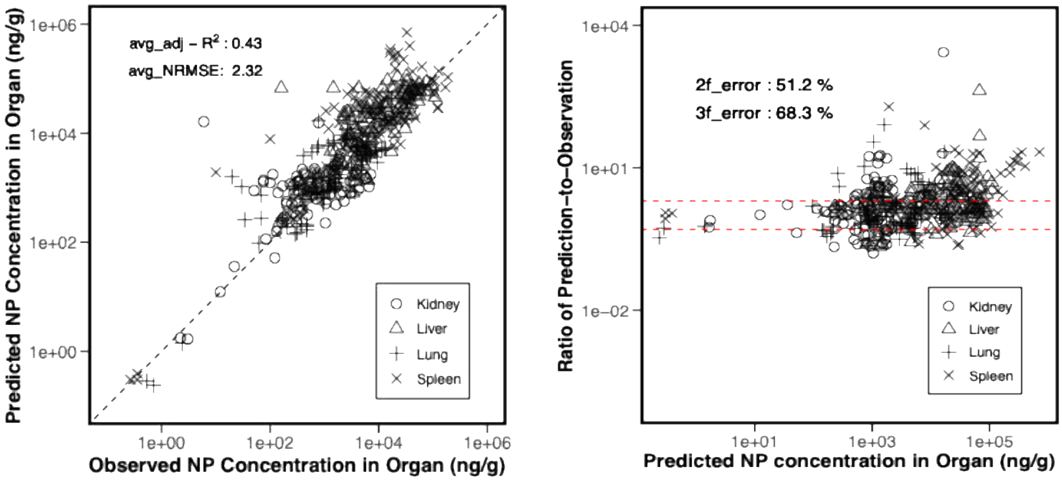

Figure S7. The accuracy of biodistribution predictions when employing PBPK model parameters derived from the multivariable linear regression model to calculate the biodistribution curve. Panel left, the solid black diagonal line represents the unity line where the observed and predicted values are equal. The figure is presented on a log-log scale since the errors are assumed to be log-normally distributed and the data span a wider range. In panel right, the dashed line represents a predicted-to-observed ratio of 2 or lower than 0.5.

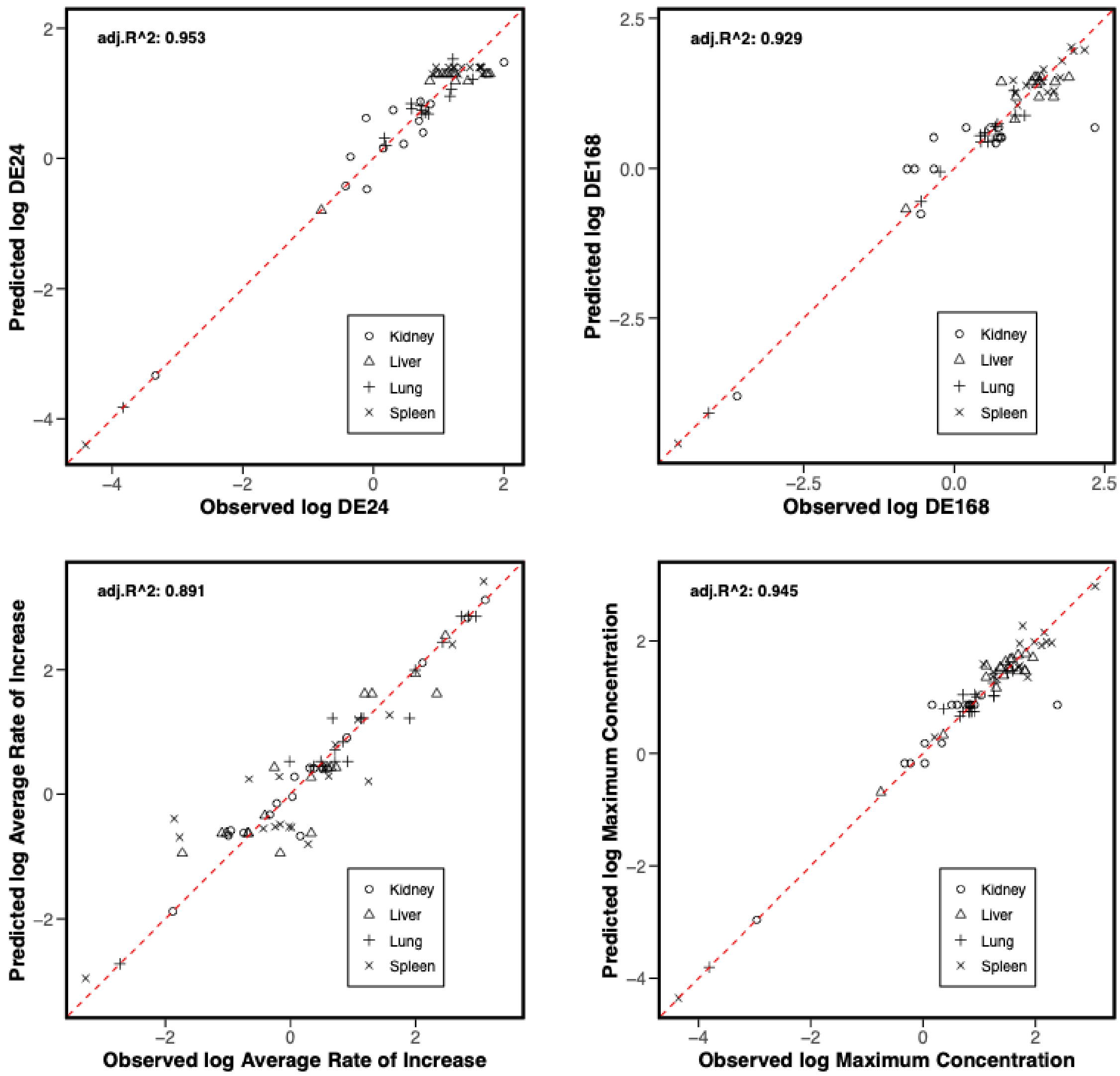

Figure S8. Evaluation of the predicted kinetic indicators generated from PBPK MLR model. A global evaluation of the goodness of model fit between the data-driven (x-axis) and PBPK MLR model-predicted delivery efficiency (DE) (y-axis) at (A) 24 h, (B) 168 h, and (C) the rate of increase (D) maximum concentration.

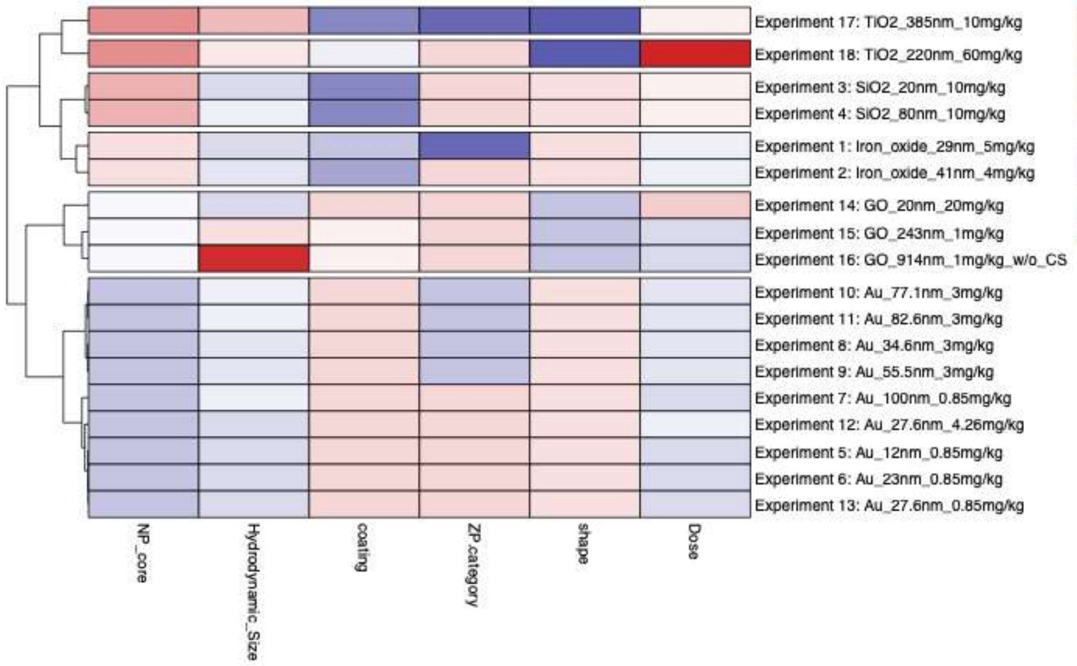

Figure S9. Heat map plot of the nanoparticle properties used for multivariable linear regression modeling. The plot identifies similarities between every nanoparticle experiment and its properties. Each column represents one property, and each row represents one nanoparticle experiment. Cell colors are coded based on the deviation of value in one experiment compared to all nanoparticle experiments for specific property, darker color represents more deviated value. Rows are sorted by hierarchical clustering (Canberra distance, ward. D2 linkage).

References

[1] B. Davies, T. Morris, Physiological Parameters in Laboratory Animals and Humans, Pharm. Res. 10 (1993) 1093–1095. https://doi.org/10.1023/A:1018943613122.

[2] R.P. Brown, M.D. Delp, S.L. Lindstedt, L.R. Rhomberg, R.P. Beliles, Physiological Parameter Values for Physiologically Based Pharmacokinetic Models, Toxicol. Ind. Health 13 (1997) 407–484. https://doi.org/10.1177/074823379701300401.

[3] L.T. Baxter, H. Zhu, D.G. Mackensen, R.K. Jain, Physiologically based pharmacokinetic model for specific and nonspecific monoclonal antibodies and fragments in normal tissues and human tumor xenografts in nude mice, Cancer Res. 54 (1994) 1517–1528.
